# Reinforcement learning-driven feature selection enhanced by an evolutionary approach tuning for criminal suspect identification

**DOI:** 10.1038/s41598-025-25920-6

**Published:** 2025-11-25

**Authors:** Zhenming Gao, Zhang Jian, Seyed Jalaleddin Mousavirad

**Affiliations:** 1 School of Management, University of Sheffield, Sheffield, UK; 2 School of Law, The University of Malaya, 50603 Kuala Lumpur, Malaysia; 3 Department of Computer and Electrical Engineering, Mid Sweden University, Sundsvall, Sweden

**Keywords:** Crime, Criminal suspects, Convolutional neural network, Proximal policy optimization, Differential evolution, Engineering, Electrical and electronic engineering

## Abstract

Accurate identification of criminal suspects is crucial for ensuring justice and deterring future crimes. Convolutional neural networks (CNNs) are frequently used to identify suspects. However, conventional methods that rely on CNNs often require assistance with feature selection (FS), class imbalance, and hyperparameter tuning, thereby diminishing their overall effectiveness. To overcome these obstacles, this study introduces a strategy based on reinforcement learning (RL), specifically off-policy proximal policy optimization (Off-policy PPO), which addresses FS and class imbalance. This approach is supplemented by a sophisticated differential evolution (DE) algorithm for tuning hyperparameters. We select Off-policy PPO because it reduces data needs, increases RL efficiency, and suits settings where data collection is costly. In our research, Off-policy PPO is dynamically tuned to improve FS and class balance. It consistently surpasses conventional static approaches by refining its approach to the intricate dynamics of criminal suspect detection. Furthermore, the DE algorithm is enhanced with a novel mutation strategy that employs k-means clustering to effectively identify key clusters. Our methodology is evaluated using four distinct datasets: the CelebFaces Attributes (CelebA), Labeled Faces in the Wild (LFW), Chinese Academy of Sciences Institute of Automation WebFace (CASIA-WebFace), and Visual Geometry Group Face 2 (VGGFace2) datasets. The experimental outcomes are remarkable, achieving F-measures of 89.409%, 91.152%, 92.184%, and 92.202%, respectively. These results demonstrate that the approach outperforms existing methods and advances early suspect detection, while also improving investigative strategies.

## Introduction

Identifying criminals is critical and challenging for law enforcement agencies, mainly due to the complexity and time-intensive nature of tracking suspects across various locations^1^. This difficulty is compounded in urban areas or crowded public spaces where population density can hinder effective surveillance. While manual identification methods can sometimes provide additional insights into criminal activities, they are not always efficient or feasible^2^. This challenge highlights the need for an automated facial recognition system that overcomes the limitations of manual methods and enhances the accuracy and efficiency of suspect identification in real-world law enforcement. This challenge underscores the need for an automated facial recognition system that overcomes the limitations of manual methods. Such a system would enhance the accuracy and efficiency of suspect identification in real-world law enforcement.

In recent years, deep learning (DL) models, particularly CNNs, have brought remarkable advancements to the task of criminal suspect recognition^3,4^. Despite these achievements, several persistent issues limit their overall performance. CNNs are powerful in capturing visual patterns. However, they often struggle with high-dimensional data that contains irrelevant or redundant features, leading to reduced classification precision. Moreover, many current frameworks have been built on highly imbalanced datasets, where minority categories (e.g., actual suspects) are underrepresented, resulting in biased predictions. An additional obstacle lies in the tuning of hyperparameters; inadequate or manually selected configurations significantly degrades detection performance and increase computational costs. To address these limitations, this research integrates Off-policy PPO and an enhanced DE algorithm, offering a comprehensive solution to the challenges of FS, class imbalance, and hyperparameter optimization (HO). By integrating these strategies, our approach effectively alleviates the weaknesses of prior techniques, enhancing accuracy, robustness, and scalability for real-world suspect identification.

Traditional FS methods, such as gradient boosting machines (GBM), random forest (RF), and decision tree (DT), have been widely used in pattern recognition tasks, including criminal suspect identification^5^. However, these methods often encounter difficulties due to the high-dimensional and heterogeneous nature of facial image data. These methods often fail to capture complex nonlinear relationships among facial attributes. Such relationships are essential for achieving accurate suspect identification. Moreover, these techniques are prone to overfitting when trained on small or imbalanced datasets, resulting in weak generalization in practical scenarios. Recently, advanced approaches such as attention mechanisms^6^, least absolute shrinkage and selection operator (LASSO)^7^, mutual information-based selection^8^, and correlation-based FS (CFS)^9^ were introduced to improve feature prioritization. While these methods have shown promise in identifying relevant features, they still have significant limitations. Attention mechanisms may focus too narrowly on localized features, ignoring broader facial patterns. LASSO and CFS can exclude valuable features because of strict penalization or threshold criteria. Mutual information-based techniques can be computationally expensive when applied to large datasets. They may also fail to capture subtle higher-order feature interactions, which limits their effectiveness in real-time suspect identification^10^.

To address class imbalance, corrective strategies have been applied at both the data level and the algorithmic level. At the data level, balancing has been achieved by oversampling minority classes or reducing the number of instances in the majority class. For instance, the synthetic minority oversampling technique (SMOTE)^11^ generates new suspect samples by interpolating between existing minority images. In contrast, NearMiss^12^ reduces the majority class samples using nearest neighbor selection. Despite their effectiveness, oversampling can cause overfitting, and undersampling may discard valuable information. On the algorithmic side, cost-sensitive learning, ensemble strategies, and decision threshold adjustments have been commonly employed^13^. Cost-sensitive learning has been used to increase penalties for misclassifying suspect images by adjusting the weights of the loss function. Ensemble methods, such as combining CNN-based classifiers, improve predictive accuracy through voting or aggregation. Threshold tuning further refines classifier outputs to better handle imbalance. However, cost-sensitive methods need precise cost calibration. Ensemble approaches can be computationally expensive. Threshold adjustments must also preserve overall accuracy while avoiding bias toward minority classes.

To overcome the limitations of traditional FS and class imbalance approaches, deep reinforcement learning (DRL) has introduced an adaptive and reward-driven framework. Unlike static methods such as GBM or LASSO, DRL can dynamically identify the most relevant features by rewarding attributes that improve classification accuracy^14^. At the same time, it filters out irrelevant or redundant data. This capability enables the model to capture complex, non-linear facial patterns that conventional methods often overlook. For class imbalance, DRL can provide a more effective solution compared to oversampling and cost-sensitive learning. DRL can assign higher rewards to correctly identified minority samples. This approach can increase sensitivity to underrepresented classes without duplicating data or causing overfitting. The adaptive learning strategy of DRL can also reduce reliance on handcrafted balancing techniques. It can help mitigate the overfitting issues seen in oversampling-based methods^15^. However, DRL models typically face difficulties concerning the bias-variance tradeoff and the need for precise hyperparameter adjustments.

PPO is an advanced on-policy RL algorithm. It can alleviate bias-variance tradeoff issues by employing a clipping mechanism that stabilizes policy updates and prevents large deviations, thereby improving training stability^16^. PPO is computationally efficient and well-suited for tasks involving continuous and complex data. Off-policy PPO can improve sample efficiency by using historical data from replay buffers. This enables the model to learn from past interactions, rather than relying solely on new samples. This approach can enhance adaptability, accelerate convergence, and facilitate more effective strategy exploration. These features can make it effective for dynamic and data-intensive tasks, such as identifying criminal suspects^17^.

Hyperparameter tuning in DRL is both important and challenging. Several optimization methods, including exhaustive search and evolutionary algorithms, have been proposed to address this issue^18^. An exhaustive search evaluates every possible combination within a predefined grid to find the optimal configuration^19^. However, this approach is time-consuming and computationally expensive. In contrast, evolutionary algorithms can improve hyperparameters using principles of natural selection and progressively evolve candidate solutions. These algorithms, however, sometimes converge slowly or risk being trapped in local optima. DE is an effective alternative. It utilizes differential vectors to update candidate solutions, thereby accelerating convergence and enhancing search efficiency. DE operates through three key steps: mutation, crossover, and selection. In the mutation step, new candidates are generated by adding scaled differences between random individuals to introduce diversity. These mutated vectors are then combined with existing solutions during crossover to enhance variation. Ultimately, in the selection phase, the most promising candidates are chosen to advance to the next generation. This iterative process strikes a balance between exploration and exploitation, preventing stagnation and navigating complex solution spaces. These characteristics can make DE suitable for HO in DRL^20^.

This paper presents a groundbreaking model for suspect identification, integrating an Off-policy PPO strategy for FS and class imbalance management, along with an advanced DE algorithm for optimizing hyperparameters. The model harnesses CNNs and multi-layer perceptrons (MLP) as its policy network, which is trained using Off-policy PPO with a tailored reward function designed to emphasize crucial features and counteract class imbalances. To refine hyperparameters, we utilize the Random Key approach, which is enhanced by a sophisticated DE algorithm that incorporates principles of the human mental search (HMS) strategy. This approach clusters the population using the k-means algorithm and selects the optimal candidate from the cluster with the lowest average objective function value for mutation. Table 1 lists the acronyms and their respective definitions used in this research.

**Table 1:** Overview of acronyms and definitions employed in the study.

Acronym	Definition
CNN	Convolutional neural network
FS	Feature selection
RL	Reinforcement learning
Off-policy PPO	Off-policy proximal policy optimization
DE	Differential evolution
CelebA	CelebFaces Attributes
LFW	Labeled Faces in the Wild
CASIA-WebFace	Chinese Academy of Sciences Institute of Automation WebFace
VGGFace2	Visual Geometry Group Face 2
DL	DL
HO	Hyperparameter optimization
GBM	Gradient boosting machines
RF	Random forest
DT	Decision tree
LASSO	Least absolute shrinkage and selection operator
CFS	Correlation-based FS
SMOTE	Synthetic minority oversampling technique
DRL	Deep reinforcement learning
MLP	Multi-layer perceptrons
HMS	Human mental search
NLP	Natural language processing
ML	Machine learning
EU SPIRIT	Scalable Privacy-preserving Intelligence analysis for Resolving Identities in real-time
MATLAB	Matrix laboratory
FAHP	Fuzzy analytic hierarchy process
CCTV	Closed-circuit television
PCA	Principal component analysis
DenseNet-169	Dense convolutional network with 169 layers
ReLU	Rectified linear unit
MTCNN	Multi-task cascade neural network
DNVPT	Deep neural vision processing techniques
WNN	Wavelet neural network
LSTM	Long short-term memory
DNN	Deep neural network
AE	Autoencoder
YOLOv8	You Only Look Once version 8
FaceNet	Facial Recognition Network
VGGFace	Visual Geometry Group Face
GhostFaceNets	A lightweight architecture designed for efficient edge-device deployment
GJO-ANN	Golden Jackal optimized artificial neural network
GAN	Generative adversarial network
DS-AEAN	Dual-scale adaptive efficient attention network
EAOA	Enhanced addax optimization algorithm
TRPO	Trust region policy optimization
KL	Kullback–Leibler
GPBA	Generic population-based algorithm
G-means	Geometric mean
AUC	Area under the curve
TP	True positive
TN	True negative
FP	False positive
FN	False negative
GB	Gigabyte
RAM	Random-access memory
CUDA	Compute unified device architecture
cuDNN	Compute unified device architecture DNN library
GPU	Graphics processing unit
RTX	Ray tracing Texel extreme
Ti	Titanium
MISSL	Multi-input spatio-structural learning
CNBA	Criminal network-based architecture
DL-ACO	DL with ant colony optimization
DCNN	Deep CNN
FECNN	Facial expression-based CNN
CLSTM	CNN-LSTM hybrid model
QN-FR	Quantum networking face recognition
YOLOv8-FI	YOLOv8-based forensic identification
FVG-FR	FaceNet-VGG-GhostFaceNet recognition
QWE-DNN	Quality-weighted embedding with DNN
FacialCueNet	Facial cues network
GAN-DSAEAN	GAN with dual-scale adaptive efficient attention network
FLOP	Floating-point operations
ITPS	Inference time per sample
RTB	Real-time bidding
FGSM	Fast gradient sign method
mRMR	Minimum redundancy maximum relevance
MI	Mutual information
TSFS	Teacher‑student FS (TSFS)
A‑SFS	Batch‑attention‑based self‑supervision FS (A‑SFS)
ROC	Receiver operating characteristic
PR	Precision-recall
SHAP	Shapley additive explanations
BO	Bayesian optimization
SSA	Salp swarm algorithm
COA	Cuckoo optimization algorithm
FA	Firefly algorithm
BA	Bat algorithm
ABC	Artificial bee colony

The main contributions of this study are outlined as follows:

- A key contribution of this study is the implementation of Off-policy PPO to improve FS. Unlike traditional static approaches, Off-policy PPO adaptively focuses on the most informative features during training. At the same time, it filters out noisy or irrelevant data. This adaptive mechanism is highly effective for the high-dimensional and complex data in criminal suspect datasets. It ensures better accuracy and efficiency in suspect identification.
- Another major contribution lies in using Off-policy PPO to mitigate class imbalance in criminal suspect identification. Off-policy PPO, through its RL framework, prioritizes minority classes by assigning higher rewards. This approach enhances sensitivity and classification accuracy for these classes. It prevents bias toward majority classes and increases the robustness of the model in real-world scenarios where suspect data is often imbalanced.
- The third contribution is the integration of an improved DE algorithm for efficient HO. This enhanced DE employs a novel mutation strategy based on k-means clustering to identify optimal parameter clusters more effectively. Automating the tuning process reduces manual effort, boosts model stability, and ensures optimal performance. The k-means-driven exploration within DE allows a more systematic and comprehensive search of the parameter space. This results in better model configurations.

The structure of this paper is methodically divided into several sections for enhanced clarity: Section “Related works” reviews pertinent literature in the field. Section “The proposed model” offers an in-depth examination of our innovative approach, detailing the primary techniques employed. Section “Empirical evaluation” outlines the outcomes of our data analysis and explores their significance. Finally, Section “Conclusion” concludes with a summary of the principal discoveries and proposes directions for further investigation.

## Related works

AI has become an essential tool across diverse fields^21,22^, such as natural language processing (NLP)^23,24^, healthcare^25,26^, and criminal prediction^27^. The ability to extract patterns from large-scale, unstructured data makes AI particularly valuable in identifying criminal suspects. For criminal suspect identification, AI analyzes large and diverse data sources, including surveillance video, biometric records, and behavioral data, to identify subtle correlations. With advanced FS, pattern recognition, and predictive modeling, AI helps law enforcement improve accuracy, shorten investigations, and reduce false identifications, which increases the reliability of forensic analysis. Prior work falls into two groups: machine learning (ML) methods that rely on feature engineering, and deep learning (DL) methods that use neural networks to learn complex patterns automatically.

### Machine Learning (ML)

ML techniques contributed to automating the identification of criminal suspects. These methods achieve this by enabling data-driven analysis of relationships among individuals, cases, and behavioral attributes. Recent studies show a variety of approaches, including network-based models, classical ML algorithms, behavioral evidence analysis, and hybrid models that combine visual analysis with optimization strategies.

The first group includes network-based models, which aim to capture the complex relationships between crime cases and individuals. Jhee et al.^28^ proposed a criminal network-based predictive framework to handle the complexities of interrelated criminal cases. This framework used a ‘sandwich panel’ structure, in which one panel represents crime cases and the opposite panel represents individuals involved, such as victims, criminals, and witnesses. They developed a fast inference algorithm to efficiently process large datasets, addressing the slow performance typically seen in network-based ML applications during real-time crime scene analysis. Jhee et al.^29^ developed a fast inference algorithm for a large-scale criminal network that featured a unique “sandwich panel” structure that connected networks of crime cases and individuals, such as victims, criminals, and witnesses. This structure was designed to efficiently manage complex connections across cases and individuals, which supported urgent criminal investigations.

The second group focuses on classical ML and clustering approaches, where algorithms are used for classification and pattern recognition. Kazemian and Shrestha^30^ applied ML techniques to an anonymized Scalable Privacy-preserving Intelligence analysis for Resolving Identities in real-time (EU SPIRIT) Horizon 2020 policing dataset to identify fraudulent identities and aid law enforcement agencies. They enhanced model accuracy using 39 million records and employed techniques such as TensorFlow with Keras, support vector machine, Naïve Bayes, and k-nearest neighbors. Before training, they optimized the model by incorporating string-matching techniques such as the Levenshtein edit distance and Jaro-Winkler to detect five suspected fraudulent identities. Kovalchuk et al.^31^ introduced an analytical method within the intelligent criminal justice framework that used k-means clustering to analyze 13,010 prisoner records from Ukraine. They identified indicators like the number of previous convictions, age at first conviction, and the presence of conditional convictions and early releases, which predicted criminal recidivism. This model supported crime investigation, court process automation, and the identification of potential repeat offenders. Sehgal et al.^32^ applied ML to analyze large datasets from criminal investigations. It enhanced real-time criminal face detection using DT algorithms and face pattern analysis tools in matrix laboratory (MATLAB). This method utilized actual criminal images to enhance face detection algorithms, significantly improving real-time functionality and predictive accuracy.

The third group includes behavioral and linguistic analysis models, which use behavioral evidence and descriptive attributes for suspect profiling. Jalal et al.^33^ developed a linguistic description-based method for suspect face image retrieval, which overcame the limitations of traditional sketch-based identification. Using the fuzzy analytic hierarchy process (FAHP), this approach assessed attribute saliency and computed weighted scores for image retrieval, which offered a sophisticated alternative for rapid suspect identification. Gupta et al.^34^ developed an automated method for suspect identification, which utilized a contrastive learning paradigm that is optimized in real-time based on user feedback. Validated through simulations and a user study, this method enhanced personalization, accelerated convergence, and improved the relevance of recommendations. It was designed for metropolitan crime investigation departments and included a user-friendly web interface for effective suspect retrieval. Barkhashree and Dhaliwal^35^ introduced an expert model for standardized behavioral evidence analysis to enhance criminal investigations by analyzing the behavioral parameters of suspects. The model extracted and linked behavioral data from various sources, including social media, and integrates demographics to deepen the understanding of suspect behaviors. It also tested ten different ML strategies to improve investigative methods.

The fourth group focuses on hybrid visual and optimization-driven approaches, where visual data analysis is combined with FS. Sivanagireddy et al.^36^ developed a sophisticated DL model designed explicitly for the precise identification of criminals from closed-circuit television (CCTV) footage. The model progressed through five phases: data collection, pre-processing, feature extraction, FS, and classification, using Haar cascade for image transformation, principal component analysis (PCA) for extraction, and ant colony optimization for FS, with classification done via the dense convolutional network with 169 layers (DenseNet-169) classifier in Pytorch.

### Deep Learning (DL)

DL has emerged as a powerful approach for identifying criminal suspects. It provides advanced feature extraction, high-level representation learning, and robust classification methods. Research in this field can be categorized into four areas: CNN-based facial recognition and detection models, hybrid and recurrent architectures, object detection using pre-trained models, and specialized or multimodal DL systems.

The first group focuses on CNN-based models. CNNs are widely used in this group for extracting facial features and performing classification tasks. Munusamy and Senthilkumar^37^ developed a facial recognition system that employed convolutional and dense layers for feature extraction and classification. The system utilized rectified linear unit (ReLU) activation functions and dropout layers to prevent overfitting, as well as max pooling layers to reduce spatial dimensions. It was tested on a customized dataset of 4288 photos to validate its effectiveness. Kumar et al.^38^ developed a real-time face detection and recognition system using a multi-task cascade neural network (MTCNN) for criminal identification. It utilized one-shot learning from a single image to detect and identify criminals, which enhanced detection efficiency. Sunday et al.^4^ enhanced crime suspect identification systems using advanced deep neural vision processing techniques (DNVPT). They applied discrete wavelet transform, rendering models, and CNNs within a MATLAB environment, and validated these implementations using tenfold cross-validation. This system was deployed in the Nigerian Police Force, demonstrating its ability to handle a diverse range of facial expressions. James et al.^39^ proposed a DL model using CNNs to predict criminal tendencies from facial expressions. The model incorporated advanced image processing techniques and integrated detailed facial features. It utilized eight convolutional layers and was fine-tuned using random search and the Adam optimizer through the Keras tuner library. This enhanced its ability to differentiate between criminal and non-criminal images.

The second group highlights hybrid and recurrent architectures, which combine CNNs with sequential models to improve performance. Lei and Huang^2^ utilized a wavelet neural network (WNN) to predict criminal suspect features that enhanced its performance with Morlet and Mexican Hat functions. The study involved preprocessing data and conducting simulation experiments to establish a robust evaluation index for the WNN in crime analysis and prediction. Shree et al.^40^ introduced a novel lightweight model for suspect identification that combined a CNN with long short-term memory (LSTM) to form a feature-recurrent system. This model synthesized diverse images and extracted facial features with fewer trainable parameters than traditional models. This resulted in showing significant accuracy improvements across multiple datasets. Raghav et al.^41^ explored a criminal identification system using deep neural networks and ML, which focused on face recognition through CCTV. This research addressed the challenge of identifying criminals who leave minimal physical evidence, which offered a more effective solution through the use of advanced facial recognition technology.

The third group includes object detection and pre-trained models. These models utilize modern detection architectures and transfer learning techniques. Sandhya et al.^1^ proposed an intelligent criminal detection system utilizing a deep neural network (DNN) model, which integrated a single-shot Multibox detector and an autoencoder (AE). The system compared facial images with a criminal database using the cosine similarity metric to enhance the accuracy of suspect tracking and identification. A confidence threshold of 0.75 was set for the encoder model to ensure reliable identification. Serka et al.^42^ utilized state-of-the-art pre-trained You Only Look Once version 8 (YOLOv8) object recognition models to enhance suspect identification in digital forensics. They trained models of varying capacities on the Wider-Face dataset, which optimized the image and video identification process. This method provided digital forensic experts with a desktop application for real-time image analysis, which streamlined the process of identifying and classifying suspects. Ardiawan et al.^43^ assessed the performance of cutting-edge facial recognition technologies, such as FaceNet (Facial Recognition Network), VGGFace (Visual Geometry Group Face), and GhostFaceNets (a lightweight architecture designed for efficient edge-device deployment). Using 2023 data, the study highlighted the superior accuracy of FaceNet, due to its triplet loss optimization and Euclidean space mappings, and compared it with other models to identify areas for improvement. Ribeiro et al.^44^ proposed a forensic facial comparison framework. The framework aggregated DNN embeddings from multiple images of the same individual and uses quality-weighted embedding fusion to enhance facial matching under uncontrolled conditions.

The final group focuses on specialized or multimodal DL systems, which integrate multiple data modalities or specialized neural architectures. Nam et al.^45^ introduced a multimodal network for deception detection in criminal interrogations, utilizing DL technology without biosensors. This system integrated facial cues and employed a spatial–temporal attention module to enhance data interpretability, trained and evaluated on real and publicly available datasets. Natarajan et al.^46^ used DL to generate face sketches for crime scene suspect prediction with the Golden Jackal optimized artificial neural network (GJO-ANN). It improved suspect identification by comparing sketches to eyewitness and artist renditions. Alzubi et al.^47^ introduced a DL-based masked face identification framework that combined a generative adversarial network (GAN), a dual-scale adaptive efficient attention network (DS-AEAN), and an enhanced addax optimization algorithm (EAOA) to handle both masked and mask-free face recognition tasks.

### Differences compared to the proposed model

Tables 2 and 3 summarize recent state-of-the-art ML and DL methods for criminal suspect detection, along with their key advantages and limitations. While these methods show considerable potential, their effectiveness and broader applicability still require enhancement. Traditional approaches often lack effective FS mechanisms. This leads to the inclusion of irrelevant or redundant features that obscure meaningful patterns and reduce model accuracy. Additionally, the inherent class imbalance in criminal datasets typically results in models biased toward the majority class, underrepresenting minority classes that are often of greater interest. Finally, HO remains a significant challenge, as conventional methods rely on extensive manual tuning, which is both time-consuming and prone to error.

**Table 2:** Comparison of ML models for criminal suspect detection.

Author	Method	Advantage	Disadvantage
Jhee et al.^28^	Criminal network-based predictive framework with a sandwich panel structure	Developed a fast inference algorithm to efficiently process large datasets, which addressed slow performance in real-time crime analysis	May struggle with the integration and processing of heterogeneous data types in real-time
Jhee et al.^29^	Developed a fast inference algorithm for a large-scale criminal network with a unique structure	Efficient management of complex connections across crime cases and individuals, which supported urgent investigations	Requires extensive computational resources for scalability
Kazemian and Shrestha ^30^	Applied ML to a large policing dataset for fraud detection	Enhanced model accuracy using 39 million records, which employed advanced techniques like TensorFlow and a support vector machine	Optimization is dependent on the initial data quality and completeness
Kovalchuk et al.^31^	Analytical method using k-means clustering on prisoner records for criminal justice	Identified predictive indicators of criminal recidivism, which supported crime investigation automation	Limited predictive power for dynamic criminal behavior outside the studied demographic
Sehgal et al.^32^	ML with DT for criminal face detection in MATLAB	Enhanced real-time face detection using actual criminal images for improved accuracy	Performance may decline with low-quality images
Jalal et al.^33^	Linguistic description-based method for suspect face image retrieval	Uses the FAHP to enhance suspect identification from sketches	Depends heavily on the accuracy and detail of linguistic descriptions and sketches
Gupta et al.^34^	Automated suspect identification method using a contrastive learning paradigm	Optimized based on real-time user feedback, included a user-friendly web interface for metropolitan crime investigations	Effectiveness may diminish without sufficient training data or in diverse operational environments
Barkhashree and Dhaliwal ^35^	Expert model for standardized behavioral evidence analysis	Analyzed behavioral parameters from various data sources, which integrated multiple ML strategies	May face challenges in correlating diverse behavioral data to specific criminal activities
Sivanagireddy et al.^36^	DL model for criminal identification from CCTV in Pytorch	Followed a comprehensive five-phase process with advanced algorithms for precise identification	Dependent on the quality of the initial data collection

**Table 3:** Comparison of DL models for criminal suspect detection.

Author	Method	Advantage	Disadvantage
Munusamy and Senthilkumar ^37^	Facial recognition system using convolutional and dense layers in MATLAB	Enhanced real-time face detection with multi-layer non-linearity	Requires a large custom dataset for validation
Kumar et al.^38^	Face detection and recognition system using an MTCNN	Employed one-shot learning for real-time, accurate criminal identification	Dependent on the quality and angle of the input images
Sunday et al.^4^	Advanced facial recognition using multiple models in MATLAB	Implemented diverse models for robust face detection under varied conditions	Limited by the processing capabilities of MATLAB for real-time analysis
James et al.^39^	CNN model to predict criminal tendencies from facial expressions	Utilized detailed facial features for enhanced prediction accuracy	Performance is heavily influenced by the diversity of the training dataset
Lei and Huang^2^	WNN for predicting criminal suspect features	Utilized advanced functions for enhancing WNN performance in crime analysis	Limited by the complexity of the preprocessing needs
Shree et al.^40^	A lightweight model that integrated CNN with LSTM for suspect identification	Reduced model complexity while maintaining high accuracy in feature extraction	May struggle with very large or diverse datasets
Raghav et al.^41^	Criminal identification system using deep neural networks and CCTV	Leveraged extensive camera networks for a comprehensive identification system	May be less effective without high-quality CCTV footage
Sandhya et al.^1^	Smart criminal detection system using a DNN model	Combined single-shot Multibox detector with an AE for accurate matching	High reliance on the accuracy of facial recognition algorithms
Serka et al.^42^	Use of YOLOv8 in digital forensics for suspect identification	Offered a scalable solution across various computational capacities for real-time analysis	Requires extensive training data to reach optimal performance
Ardiawan et al.^43^	Evaluation of FaceNet, VGGFace, and GhostFaceNets for suspect identification	Assessed and compared multiple models to find the most accurate for facial recognition	Some models may not perform well under all conditions
Ribeiro et al.^44^	Forensic facial comparison via aggregated DNN embeddings	Improved facial matching by combining embeddings from multiple images, which reduced the impact of low-quality or uncontrolled conditions	Relies on the availability of multiple images for effective aggregation
Nam et al.^45^	Multimodal network for deception detection without biosensors	Integrated facial cues with a spatial–temporal attention module for improved interpretability	May lack robustness in uncontrolled environments
Natarajan et al.^46^	DL model for face sketch synthesis for crime scene suspect prediction	Automated suspect identification by comparing sketches with real images	Effectiveness may vary based on sketch quality and detail
Alzubi et al.^47^	GAN-based masked face identification with DS-AEAN and EAOA	Handled both masked and mask-free facial images using GAN data generation and attention-based feature extraction	The complexity of architecture and optimization may lead to high computational cost

To solve these problems, this article introduces a comprehensive approach that synergistically combines advanced ML techniques. By integrating Off-policy PPO, this study directly addresses the dual challenges of FS and class imbalance within the learning process. Off-policy PPO is utilized to dynamically adjust feature weights and selection criteria in response to ongoing learning, ensuring only the most predictive features are utilized. This adaptability makes it particularly suitable for complex, imbalanced datasets typical in criminal suspect detection. Moreover, incorporating a DE algorithm for hyperparameter tuning automates this process, reducing the need for manual intervention and enhancing the overall robustness and reliability of the model. This DE algorithm incorporates a novel mutation strategy utilizing k-means clustering, which efficiently identifies and optimizes key parameters to ensure optimal performance across diverse datasets. Together, these innovations provide a powerful solution to the previously mentioned limitations, significantly improving the detection and identification of criminal suspects with higher accuracy and efficiency.

## The proposed model

**Fig. 1:**
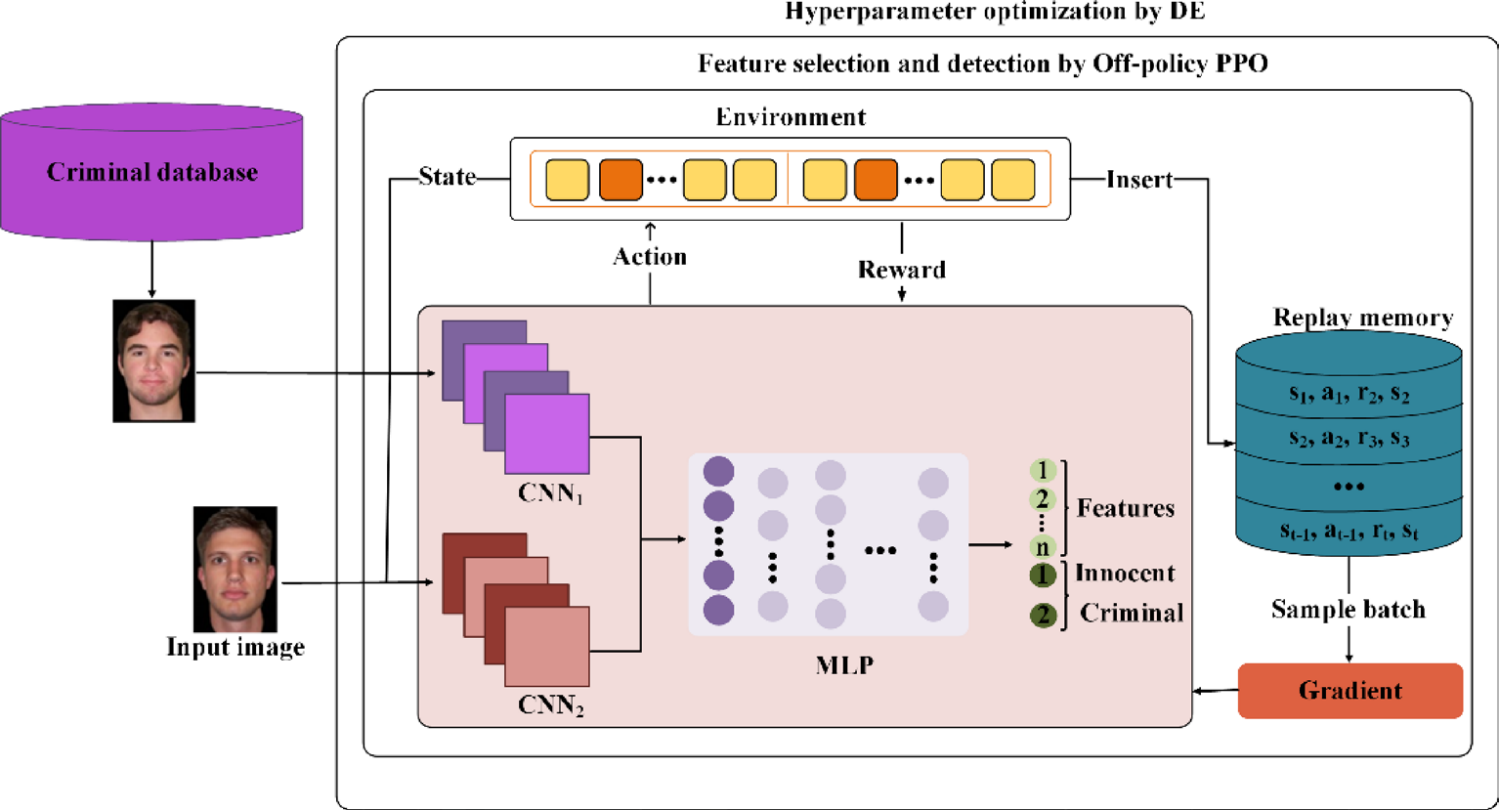
Architecture of the proposed criminal suspect prediction model. It includes a two-stream CNN for feature extraction, FS and class imbalance via Off-policy PPO, and HO using the DE algorithm.

This paper presents a DL model for criminal suspect identification. It addresses FS and class imbalance with Off-policy PPO and performs HO with a DE algorithm. Figure 1 illustrates the comprehensive model for predicting criminal suspects. The architecture comprises two distinct CNNs, one for processing the input image and another for analyzing a suspect image extracted from the dataset, along with an MLP. The procedure starts with the CNNs processing the initial image to remove unnecessary components, enhancing FS. The processed outputs from the CNNs are then channeled into an MLP network. This MLP generates $n+2$ outputs, where $n$ denotes the number of features (corresponding to the outputs from each CNN), and $2$ denotes the number of classes (innocent and criminal).

The following subsections present the mathematical formulation and algorithmic steps of the proposed model, including FS and class imbalance (via Off-policy PPO), and hyperparameter tuning (via DE).

### FS and detection

This section explores the complexities of FS and prediction in criminal suspect identification using a sophisticated RL approach, specifically Off-policy PPO. We use RL owing to its aptitude for managing complex decision-making tasks that often challenge traditional supervised methods. Unlike conventional supervised learning, which rigidly links features based on their correlations, RL dynamically adjusts FS based on contextual requirements. Essentially, RL is dedicated to refining decision-making sequences to pinpoint the most vital features, significantly enhancing the accuracy of predictions.

Consider the training dataset D, which includes pairs $(({I}_{c},{I}_{s}),y)$, where ${I}_{c}$ and ${I}_{s}$ are images of the criminal and suspect derived from feature vectors produced by CNNs, $F = \{{f}_{1}, ..., {f}_{n}\}$, and $y$ represents the target label. In each iteration, a random pair is selected from $D$. The agent then determines which features to utilize. In our RL model, the state, action, and reward dynamics are defined as follows:

- *State*: The state space $S$ in our RL model is designed to encompass each instance ((${I}_{c},{I}_{s}$), y, F), including the specific images (${I}_{c},{I}_{s}$), the corresponding label $y$, and the selected features $F$.
- *Action space*: The action space of our model $A$ includes choices to either select a feature (${A}_{f}$) or proceed to make a prediction (${A}_{c}$). Selecting a feature $a\in {A}_{f}$ leads to a state change where the set $F$ is enlarged, thus modifying the feature landscape for predictions. The related reward is $-\lambda \times c({f}_{i})$, where $c$ (${f}_{i}$) indicates the cost of adding feature ${f}_{i}$, and $\lambda$ adjusts the balance between feature cost and prediction accuracy. Actions that culminate in making a prediction (${A}_{c}$) move the model to a terminal state, with rewards or penalties of  ± 1 for the minority class (${D}_{O}$) and $\pm \gamma$ for the majority class (${D}_{N}$). The reward function $r: S\times A\to R$ is structured as follows^48^:
  1 $$r\left( {\left( {x,y,{\mathbb{F}}} \right),a} \right) = \left\{ {\begin{array}{*{20}l} { - \lambda \times c\left( {f_{i} } \right)} & { if\;a \in A_{f} ,\;a = f_{i} } \\ { + 1} & { if\;a \in A_{c} ,\;s_{t} \in D_{O} ,\;a = y} \\ { - 1} & { if\;a \in A_{c} ,\;s_{t} \in D_{O} ,\;a \ne y} \\ { + \gamma } & { if\;a \in A_{c} ,\;s_{t} \in D_{N} ,\;a = y} \\ { - \gamma } & { if\;a \in A_{c} ,\;s_{t} \in D_{N} ,\;a \ne y} \\ \end{array} } \right.$$

Following the outlined description, the state transition process of the model, denoted as $t: S\times A\to S\cup T$, operates as follows:

2 $$t\left( {\left( {x,y,{\mathbb{F}}} \right),a} \right) = \left\{ {\begin{array}{*{20}l} T & {if\;a \in A_{c} } \\ {\left( {x,y,{\mathbb{F}} \cup a} \right)} & {if\;a \in A_{f} } \\ \end{array} } \right.$$

In this framework, $T$ denotes the terminal state. When an action adds a feature, it is included in the current set $F$. The episode ends when the agent chooses the prediction action. The RL model operates in a discrete-time setting, where $t$ in ${s}_{t}$ and ${s}_{t+1}$ represents decisions made during FS. Each decision selects a feature to improve prediction accuracy. This selection affects the next state and the reward received. This sequential decision process differentiates RL from traditional supervised learning with static feature sets.

Algorithm 1 outlines the complete pseudocode for the proposed criminal suspect identification framework. Our method integrates a dedicated FS mechanism with a dual-action policy that dynamically determines whether the next step should involve selecting features or performing classification. Unlike classical PPO, which updates policies using only the current state–action pairs, the proposed approach stores full state transitions in a memory buffer $B$ to enable off-policy learning. This design enables the reuse of past experiences, which improves sample efficiency and stabilizes training. The reward function (Eq. 1) is carefully designed to emphasize minority classes and to penalize redundant FSs, thereby increasing robustness in highly imbalanced datasets.

**Algorithm 1:**
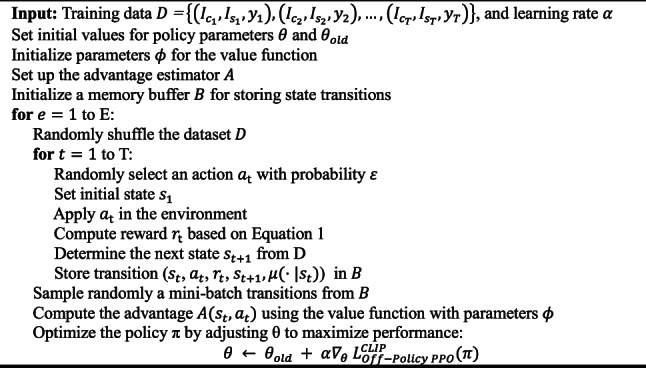
Pseudocode for the proposed criminal suspect identification model.

#### Training

To enhance policy training, an Off-policy PPO algorithm is incorporated into the RL framework. In the following, we introduce the trust region policy optimization (TRPO) method, which seeks to enhance policy performance by refining a surrogate objective using on-policy data. Subsequently, we discuss the PPO, which introduces a clipped surrogate objective to curtail the extensive policy updates often encountered in TRPO. Lastly, we present the Off-policy PPO approach, which integrates strategies from both TRPO and PPO.

##### TRPO

In RL, the objective is to find a policy $\pi$ that maximizes the expected cumulative discounted reward^17^:

3 $$\eta \left( \pi \right) = E_{{s_{0} ,a_{0} , \ldots }} \left[ {R_{0} } \right] = E_{{s_{0} ,a_{0} , \ldots }} \left[ {\mathop \sum \limits_{t = 0}^{\infty } \gamma^{t} r\left( {s_{t} ,a_{t} } \right)} \right]$$

In this formulation, ${s}_{0}\sim {\uprho }_{0}$ indicates that the initial state is drawn from a starting distribution. The policy $\pi ({a}_{t}|{s}_{t})$ defines the action probabilities in each state, and the transition function $P({s}_{t+1}{|s}_{t},{a}_{t})$ models the probability of moving to the next state given the current state and action. The discount factor $\gamma$ adjusts the importance of future rewards. TRPO updates the policy by maximizing a surrogate objective while limiting policy shifts through a Kullback–Leibler (KL) constraint^17^:

4 $$\mathop {\max }\limits_{\pi } E_{{s\sim \rho_{{\pi_{{\theta_{old} }} }} , a \in \pi_{{\pi_{old} }} }} \left[ {\frac{{\pi_{\theta } \left( {a{|}s} \right)}}{{\pi_{{\theta_{old} }} \left( {a{|}s} \right)}}A_{{\pi_{old} }} \left( {s,a} \right)} \right]$$

Subject to

5 $$E_{{s\sim \rho_{{\pi_{old} }} }} \left[ {D_{KL} (\pi_{old} (.|s)|| \pi (.|s))} \right] \le \delta$$

Here, ${\pi }_{old}$ symbolizes the current policy, and $\delta$ sets the limit for divergence. The expression ${D}_{KL}({\pi }_{old}(.|s)|| \pi (.|s))$ measures the KL divergence, assessing the extent to which the policy $\pi$ differs from the preceding policy ${\pi }_{old}$ at a given state $s$. The symbol ${\rho }_{{\pi }_{old}}$ represents the discounted state distribution that arises from the initial state ${s}_{0}$ when following the old policy ${\pi }_{old}$, calculated as ${\rho }_{{\pi }_{old}}\left(s\right)=\sum_{t=0}^{\infty }{\gamma }^{t}P({s}_{t}=s|{s}_{0},{\pi }_{old})$. Nonetheless, absent this divergence constraint, optimizing the surrogate objective function mentioned in Eq. 5 could result in extensive modifications to the policy.

##### PPO

To stabilize policy updates, PPO optimizes a clipped surrogate objective^17^:

6 $$L_{PPO}^{CLIP} = E_{{s\sim \rho_{{\pi_{old} , a\sim \pi_{old} }} }} \left[ {{\text{min}}\left( {\frac{{\pi \left( {a{|}s} \right)}}{{\pi_{old} \left( {a{|}s} \right)}}A_{{\pi_{old} }} \left( {s,a} \right),clip\left( {\frac{{\pi \left( {a{|}s} \right)}}{{\pi_{old} \left( {a{|}s} \right)}},1 - \in ,1 + \in } \right)A_{{\pi_{old} }} \left( {s,a} \right)} \right)} \right]$$

The term $\epsilon$ is a small, positive constant that balances stability and exploration. The advantage function ${A}_{{\pi }_{old}}\left(s,a\right)$ indicates how much more reward the action *a* in state *s* yields compared to the average reward of all possible actions in that state under the old policy. Clipping the ratio limits drastic policy updates and stabilizes the learning process^17^:

7 $$clip\left( {x,{ }a,{ }b} \right){ } = { }max\left( {a,{ }min\left( {b,{ }x} \right)} \right)$$

where $x$ is the value to be adjusted. $a$ is the minimum limit, and $b$ is the maximum limit of the range within which x is restricted. This design penalizes updates where the probability ratio diverges significantly from 1. Although PPO offers significant advantages, it also has a key limitation. It relies heavily on on-policy data, which leads to high sample complexity. This dependency necessitates frequent interaction between the agent and the environment, thereby increasing the computational cost of training.

##### Off-policy PPO

In this section, we introduce the Off-policy PPO algorithm, which improves sample efficiency by leveraging data from past experiences and various policy decisions. Unlike traditional on-policy PPO, which primarily uses data from current environmental interactions and requires large amounts of such data to be effective, Off-policy PPO benefits from a broader dataset. This dataset includes historical interactions and diverse strategic decisions. For instance, consider a scenario where a robot is navigating a complex maze. While traditional on-policy PPO relies solely on real-time data to adjust its strategies, Off-policy PPO leverages previous navigation attempts and makes different strategic decisions under various conditions. This method accelerates the learning process by minimizing the need for the robot to extensively explore every potential path, and enhances operational effectiveness by leveraging a richer array of experiences.

Off-policy PPO addresses the optimization challenge by aiming to maximize a surrogate objective using off-policy data, similar to the approach taken in Off-Policy TRPO^17^:

8 $$\mathop {\max }\limits_{\pi } E_{{s\sim \rho_{\mu } , a \in \mu }} \left[ {\frac{{\pi \left( {a{|}s} \right)}}{{\mu \left( {a{|}s} \right)}}A_{{\pi_{old} }} \left( {s,a} \right)} \right]$$

Subject to:

9 $$\overline{D}_{KL}^{{\rho_{\mu } ,sqrt}} \left( {\mu ,\pi_{old} } \right)\overline{D}_{KL}^{{\rho_{\mu } ,sqrt}} \left( {\pi_{old} ,\pi } \right) + \overline{D}_{KL}^{{\rho_{\mu } }} \left( {\pi_{old} ,\pi } \right) \le \delta .$$

where

10 $$\rho_{\mu } \left( s \right) = \mathop \sum \limits_{t = 0}^{\infty } \gamma^{t} P(s_{t} = s|s_{0} ,\mu )$$

11 $$\overline{D}_{KL}^{{\rho_{\mu } }} \left( {\pi_{old} ,\pi } \right) = E_{{s\sim \rho_{\mu } }} \left[ {D_{KL} \left( {\pi_{old} \left( {.{|}s} \right) || \pi \left( {.{|}s} \right)} \right)} \right]$$

12 $$\overline{D}_{KL}^{{\rho_{\mu } ,sqrt}} \left( {\mu ,\pi_{old} } \right) = E_{{s\sim \rho_{\mu } }} \left[ {\sqrt {D_{KL} \left( {\mu \left( {.{|}s} \right) || \pi_{old} \left( {.{|}s} \right)} \right)} } \right]$$

13 $$\overline{D}_{KL}^{{\rho_{\mu } ,sqrt}} \left( {\pi_{old} ,\pi } \right) = E_{{s\sim \rho_{\mu } }} \left[ {\sqrt {D_{KL} \left( { \pi_{old} \left( {.{|}s} \right) || \pi \left( {.{|}s} \right)} \right)} } \right]$$

where μ represents the behavior policy. Absent the limitation outlined in Eq. 9, the goal of maximizing the surrogate objective with off-policy data, as indicated in Eq. 8, may result in significant policy updates. To mitigate this potential issue, employing the PPO clipping technique proves beneficial by modifying the surrogate objective^17^:

14 $$L_{\mu } \left( \pi \right) = E_{{s\sim \rho_{\mu } , a \in \mu }} \left[ {\frac{{\pi \left( {a{|}s} \right)}}{{\mu \left( {a{|}s} \right)}}A_{{\pi_{old} }} \left( {s,a} \right)} \right]$$

With ${L}_{\mu }\left(\pi \right)$ in Eq. 14, the related clipped surrogate goal utilizing off-policy data is^17^:

15 $$\overline{L}_{\mu } \left( \pi \right) = E_{{s\sim \rho_{\mu } , a \in \mu }} \left[ {{\text{min}}\left( {\frac{{\pi \left( {a{|}s} \right)}}{{\mu \left( {a{|}s} \right)}}A_{{\pi_{old} }} \left( {s,a} \right),clip\left( {\frac{{\pi \left( {a{|}s} \right)}}{{\mu \left( {a{|}s} \right)}},1 - \in ,1 + \in } \right)A_{{\pi_{old} }} \left( {s,a} \right)} \right)} \right]$$

Generally, the ratio $\frac{\pi \left(a|s\right)}{\mu \left(a|s\right)}$ exceeds the limits of 1−ε and 1 + ε. Consequently, the policy $\pi \left(a|s\right)$ typically stays the same while optimizing the clipped surrogate objective. To mitigate this static effect, the limits of the clipped objective $((1 - \in ),(1 + \in ))$ are adjusted in Eq. 15 by incorporating a correction factor of $\frac{{\pi }_{{\theta }_{i}}\left(a|s\right)}{\mu \left(a|s\right)}$^17^:

16 $$\begin{aligned} & L_{Off - Policy PPO}^{CLIP} \left( \pi \right) = E_{{s\sim \rho_{\mu } , a \in \mu }} \\ & \quad \left[ {{\text{min}}\left[ {\frac{{\pi \left( {a{|}s} \right)}}{{\mu \left( {a{|}s} \right)}}A_{{\pi_{old} }} \left( {s,a} \right),clip\left( {\frac{{\pi \left( {a{|}s} \right)}}{{\mu \left( {a{|}s} \right)}},\frac{{\pi_{old} \left( {a{|}s} \right)}}{{\mu \left( {a{|}s} \right)}}\left( {1 - \in } \right),\frac{{\pi_{old} \left( {a{|}s} \right)}}{{\mu \left( {a{|}s} \right)}}\left( {1 + \in } \right)} \right)A_{{\pi_{old} }} \left( {s,a} \right)} \right]} \right] \\ \end{aligned}$$

### HO

Optimizing hyperparameters is essential in RL, as it directly impacts performance and training efficiency. Well-tuned parameters improve convergence speed, training stability, and policy effectiveness. This process enhances generalization across various tasks, reduces computational cost by eliminating unnecessary iterations, and facilitates improved performance with fewer training episodes.

Table 4 outlines the hyperparameters adjusted during our research. The value intervals are based on prior studies in DL for criminal suspect identification and define the search space used by the DE algorithm. This setup allows the algorithm to apply targeted modifications tailored to the specific demands of the proposed model.

**Table 4:** Hyperparameters and their configured values used in the proposed model.

Hyperparameter	Range	Best value
γ	[0–1]	CelebA (0.52) and LFW (0.48)
λ	[0–1]	CelebA (0.34) and LFW (0.35)
Learning rate (α)	[0–1]	CelebA (0.002) and LFW (0.001)
Activation function	[ReLU, Leaky ReLU, Tanh, Sigmoid]	ReLU
Dropout rate	[0–1]	CelebA (0.36) and LFW (0.38)
Epoch	[8–128]	CelebA (253) and LFW (274)
Batch size	[16–512]	CelebA (36) and LFW (42)
Number layers of MLP	[2–8]	CelebA (2) and LFW (4)

#### Random key

This paper employs the Random Key approach for HO due to its simplicity and effectiveness in exploring diverse configurations. The stochastic nature of the method helps avoid local optima by randomly sampling a wide range of hyperparameter values, increasing the likelihood of discovering optimal settings in complex search spaces.

The Random Key approach represents each solution as a real-valued vector ${p}_{i}$ with $D$ dimensions. This vector is divided into $C$ segments, each corresponding to a hyperparameter. Each segment contains ${D}_{c}$ values, where ${D}_{c}=1$ for continuous hyperparameters. The total dimensionality $D$ is the sum of all ${D}_{c}$ values. For categorical hyperparameters, the segment is transformed into a category using a mapping function ${MAP}_{c}$. The values in the segment are ranked, and the highest-ranked value determines the selected category. This encoding enables the direct application of evolutionary operations, mutation, crossover, and selection to ${p}_{i}$, allowing for efficient global optimization across both continuous and discrete spaces. The method simplifies the encoding process, supports diverse search strategies, and is particularly effective in high-dimensional or poorly understood hyperparameter spaces.

**Fig. 2:**
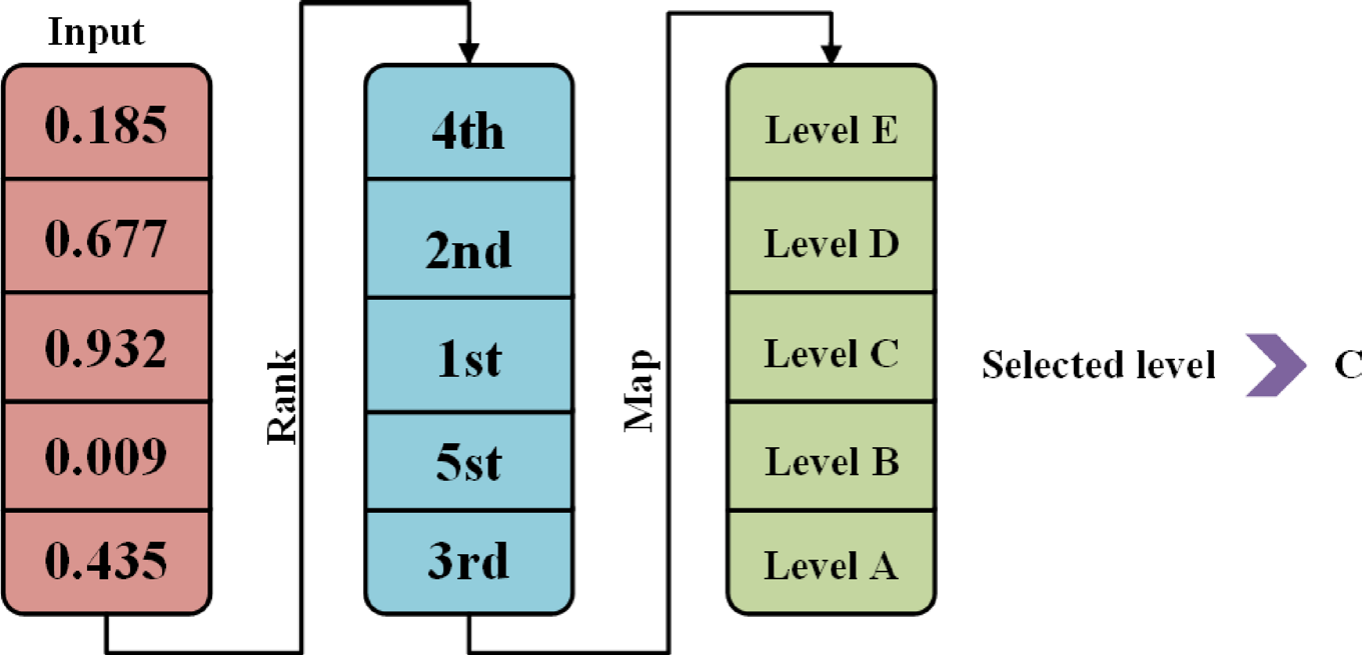
Example of the Random key method with ${D}_{c}$ = 5 for HO.

An example is the ‘number of layers’ hyperparameter, set at ${D}_{c}$ = 5, illustrated in Fig. 2. Essentially, the random key sorts real numbers according to their magnitudes. This sorting is vital for matching vectors with designated option arrays. Over time, this system allows key attributes to become more prominent within the key, thereby enhancing the efficiency of the model in evaluating and prioritizing feature significance.

#### DE

To improve the Random Key method, we utilize the DE algorithm due to its effectiveness in solving non-linear and multimodal problems. DE applies mutation and crossover strategies to increase population diversity and guide convergence toward the global optimum. This integration improves the exploration and exploitation of the search space. As a result, it enables more accurate HO and boosts the performance and reliability of the Random Key method in complex classification tasks.

The DE algorithm operates through three key steps: mutation, crossover, and selection. In the mutation stage, the algorithm creates a new candidate solution by selecting two individuals from the population, calculating the difference between them, scaling this difference, and adding it to a third base vector. This step maintains diversity and helps avoid premature convergence. The crossover phase then combines the mutated vector with a target vector to explore new regions, while the selection phase retains the better-performing solution. These steps collectively guide the search toward globally optimal hyperparameters.

In DE, the mutation process generates a new candidate vector as described below:

17 $${\overrightarrow{v}}_{i,g}= {\overrightarrow{x}}_{{r}_{1},g}+F ({\overrightarrow{x}}_{{r}_{2},g}- {\overrightarrow{x}}_{{r}_{3},g})$$

In this phase, the algorithm randomly selects three candidate vectors ${\overrightarrow{x}}_{{r}_{1},g}$, ${\overrightarrow{x}}_{{r}_{2},g}$, and ${\overrightarrow{x}}_{{r}_{3}}$. The difference between the two of them is scaled by a factor $F$ and added to the third to create a mutant vector. This vector is then combined with a target vector using the binomial crossover, enhancing the diversity and exploratory capacity of the population:

18 $$u_{i,j,g} = \left\{ {\begin{array}{*{20}l} {v_{i,j,g} } & {if\;rand\left( {0,{ }1} \right) \le CR\;or\;j = j_{rand} } \\ {x_{i,j,g} } & {otherwise} \\ \end{array} } \right.$$

In this step, the crossover rate $CR$ and a randomly chosen index ${j}_{rand}$ from the range $\{\text{1,2},...,D\}$ control how the vectors are combined, where $D$ is the total number of dimensions. In the selection stage, the algorithm compares the original vector and the trial vector. The one with better performance is retained, which helps maintain solution quality and encourages ongoing improvement.

To enhance the performance of DE, we use an improved version that incorporates a novel mutation strategy^49^. The process begins by implementing k-means clustering on the existing population to identify distinct segments within the search space. This division results in several clusters, with the number of clusters, k, being randomly determined from a range between $[2, \sqrt{N}]$, where $N$ is the population size. Focus is then directed to the cluster with the smallest average objective function value, which is chosen for in-depth analysis. The mutation function, redefined through this clustering approach, is specified as follows:

The refined mutation function, shaped by the clustering process, is outlined below:

19 $$\overrightarrow{{{v}^{clu}}_{i}}= {\overrightarrow{win}}_{g}+F ({\overrightarrow{x}}_{{r}_{1},g}- {\overrightarrow{x}}_{{r}_{2},g})$$

In this method, ${\overrightarrow{x}}_{{r}_{1},g}$ and ${\overrightarrow{x}}_{{r}_{2},g}$ are two solutions randomly selected from the population, while ${\overrightarrow{win}}_{g}$ represents the best-performing solution within the most promising cluster. It is essential to note that ${\overrightarrow{win}}_{g}$ may not be the superior solution throughout the entire population, but it stands out within its respective cluster. This focus on cluster-based mutation is consistently employed over $M$ iterations to improve solution quality within the designated cluster. Following this, the population undergoes various phases as prescribed by the generic population-based algorithm (GPBA):

- *Selection*: Initially, k-candidate solutions are chosen at random to serve as the initial centers for k-means clustering.
- *Generation*: The mutation step produces $M$ new candidate solutions, collectively called the group ${v}^{clu}$.
- *Replacement*: $M$ candidates are selected at random from the larger pool to create the set $B$.
- *Update*: The top-performing $M$ candidates from the combined groups of ${v}^{clu}$ and $B$ form the new set $B{\prime}$. The population is then updated by combining the remaining members $(P-B)$ and $B{\prime}$.

**Algorithm 2:**
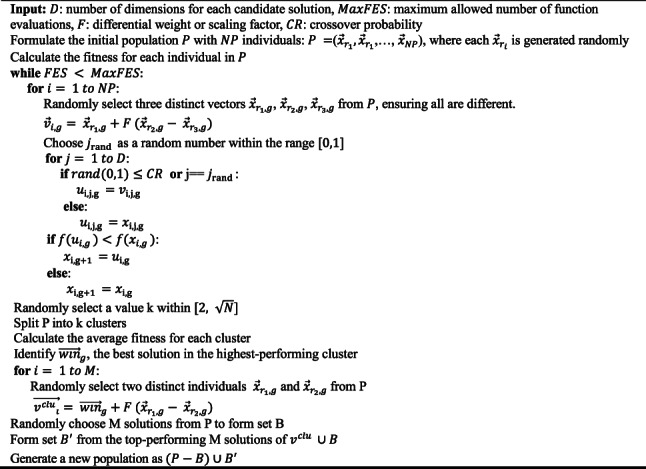
Pseudo-code for the proposed DE algorithm.

The pseudo-code of the improved DE algorithm is shown in Algorithm 2. Our approach differs from classical DE by employing a cluster-guided mutation strategy, which involves performing mutations and crossovers uniformly across the population. Specifically, we first apply k-means clustering to the population to identify distinct subgroups within the search space. The number of clusters $k$ is randomly chosen from $[2, \sqrt{N}]$. We select the cluster with the lowest average fitness for focused mutation. The best-performing individual in this cluster, denoted as ${\overrightarrow{win}}_{g}$, is used as the base vector during mutation. This modification directs the search process toward promising regions of the solution space while maintaining diversity. This cluster-based mutation is repeated for $M$ iterations to exploit the best regions locally, while global exploration is maintained through the standard DE mutation and crossover steps. These changes enhance the conventional DE framework, improving convergence and reducing the chances of stagnation.

### Time complexity analysis

The computational complexity of the proposed model is derived from three components: CNN-based feature extraction, Off-policy PPO FS and classification, and DE HO. Below, we analyze the time complexity of each component and provide an overall analysis of the complexity.

- *CNN feature extraction*: For a CNN with $L$ convolutional layers, where each layer has $f_{{\text{i}}}$ filters of size ${k}_{i}\times {k}_{i}$ applied to feature maps of size ${m}_{i}\times {m}_{i}$. The time complexity is given as:
  20 $$O\left(\sum_{i=1}^{L} {f}_{i}\times {k}_{i}^{2}\times {m}_{i}^{2}\times {c}_{i}\right)$$
  where ${c}_{i}$ represents the number of input channels in layer $i$. The additional costs from pooling and fully connected layers are small, so they can be included in the overall term. This complexity captures the cost of forward propagation for processing the input and suspect images.
- Off-policy PPO: The Off-policy PPO algorithm performs FS and classification over $E$ episodes, each with $T$ time steps. At each step, the algorithm selects features from the total of $n$ features, then computes the advantage function, and finally updates the policy network. The complexity is:
  21 $$O\left(E\times T\times \left(n+P+{P}_{v}\right)\right)$$
  where $P$ and ${P}_{v}$ denote the number of parameters in the policy and value networks, respectively. This complexity includes the cost of FS, advantage estimation, and gradient-based updates during policy optimization.
- DE-based HO: The improved DE algorithm optimizes hyperparameters by performing iterative mutation, crossover, and selection on a population of size $NP$ across $G$ generations. Each candidate solution is represented in $D$ dimensions. Additionally, k-means clustering adds a minor cost of $O({I}_{k}\times k\times NP\times D)$, where ${I}_{k}$ denotes the number of clustering iterations and $k$ refers to the number of clusters. Thus, the complexity is:
  22 $$O\left(G\times NP\times D+{I}_{k}\times k\times NP\times D\right)$$
- *Overall complexity*: When these components are combined, the total time complexity of the proposed framework can be approximated as:
  23 $$O\left(\sum_{i=1}^{L} {f}_{i}\times {k}_{i}^{2}\times {m}_{i}^{2}\times {c}_{i}+E\times T\times \left(n+P+{P}_{v}\right)+G\times NP\times D\right)$$

Among these components, the CNN feature extraction term $\sum_{i=1}^{L} {f}_{i}\times {k}_{i}^{2}\times {m}_{i}^{2}\times {c}_{i}$ typically dominates. This dominance is more pronounced with large images and deeper networks, as convolutional operations scale with both the filter size and the dimensions of the feature maps. The PPO term $E\times T\times \left(n+P+{P}_{v}\right)$ is moderate, as episodes and time steps are finite, and dynamic FS reduces the effective feature space $n$. The DE component $G\times NP\times D$ increases with population size and the number of generations. However, it remains relatively small because the global search strategy of DE is more efficient and faster than exhaustive grid search. Overall, the complexity remains manageable, especially as PPO reduces unnecessary features, while DE accelerates convergence in hyperparameter tuning, avoiding the quadratic growth common in brute-force approaches.

## Empirical evaluation

The section opens with an in-depth examination of the dataset, discussing its characteristics and relevance to our study. It progresses to a review of the metrics used, detailing the principal standards and criteria for evaluating the performance of our model. Following this, the results are presented, highlighting the crucial outcomes of our experiments and their implications for our research goals.

### Dataset

This article uses the CelebA^34^ and LFW^1^ datasets to evaluate our proposed model for criminal suspect identification. CelebA is selected for its extensive range of facial attributes and diversity in appearance, aiding in testing the robustness of our model against varied human features. LFW is chosen for its real-world, unconstrained facial images, a rigorous benchmark for gauging the accuracy of the model in realistic scenarios. These datasets, when combined, create a comprehensive testing environment that reflects the diversity and challenges encountered in practical applications, such as enhancing security systems or supporting law enforcement in accurately identifying suspects from surveillance footage. This combination ensures that our model handles various facial orientations, expressions, and lighting conditions, which are essential for real-world criminal identification deployments.

- *CelebA*: This dataset contains 202,599 images representing 10,177 distinct identities, each annotated with 40 binary attributes, such as “pointy nose” and “wavy hair.” To align with the Criminal Dataset, which features a unique image per individual, we removed any duplicates from CelebA. The CelebA dataset offers comprehensive coverage across various ethnicities and genders, making it a valuable research resource.
- *LFW*: This dataset includes over 13,000 images of notable individuals worldwide, available for download from the LFW Face Database website of the University of Massachusetts Amherst. Offered in various formats, the primary download consists of 13,233 original images compressed into a 173 MB tar file. The images are also available in two processed formats—aligned using “funneling” and “deep funneling” techniques. LFW features images of 5749 individuals, about 1680 depicted in more than two images. The dataset comprises four primary image sets and three types of “aligned” images. We use the “deep-funneled” version, which is known for providing the highest accuracy in face verification tasks.

**Fig. 3:**
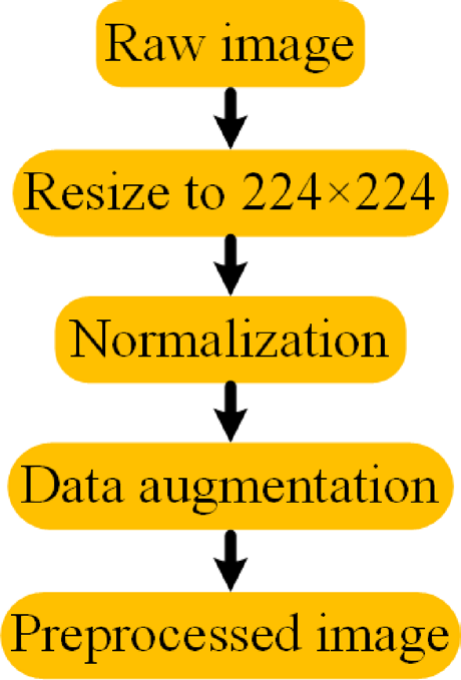
Preprocessing pipeline applied to the CelebA and LFW datasets.

Before feeding the images into the model, several preprocessing steps are applied to enhance consistency and performance. Figure 3 presents the preprocessing pipeline applied to the CelebA and LFW datasets. The process begins with resizing the raw images to 224 × 224 pixels to standardize input dimensions. Next, normalization is performed by scaling all pixel values to the range of [0, 1]. Finally, data augmentation is applied using horizontal flipping and random brightness adjustment to increase variability and improve generalizability during model training.

### Metrics

We utilize a comprehensive set of metrics, including accuracy, F-measure, geometric mean (G-means), and area under the curve (AUC)^50^, to evaluate the proposed model for criminal suspect identification. Accuracy is crucial for determining the overall effectiveness of the model in correctly identifying suspects versus non-suspects. F-measure balances precision and recall, which is essential in scenarios where false positives and negatives have serious implications. G-means is used because it effectively assesses the balance between sensitivity and specificity, which is particularly important in imbalanced datasets common in suspect identification. Lastly, the AUC measures the ability of the model to discriminate between classes across different thresholds, which helps understand the performance of the model at various levels of decision criteria, ensuring robustness in diverse operational environments.

The accuracy, F-measure, and G-means metrics are defined as follows:

24 $$Accuracy = \frac{{True\;Positives\;\left( {TP} \right) + True\;Negatives\;\left( {TN} \right)}}{Total\;observations}{ }$$

25 $$F{ - }measure \left( {F1\;Score} \right) = 2 \times \frac{Precision \times Recall}{{Precision + Recall}}{ }$$

26 $$G{ - }means = \sqrt {Recall \times Specificity}$$

where

27 $$Precision = \frac{TP}{{TP + False\;Positives \left( {FP} \right)}}{ }$$

28 $$Recall = \frac{TP}{{TP + False\;Negatives \left( {FN} \right)}}{ }$$

29 $$Specificity = \frac{TN}{{TN + FP}}{ }$$

### Results

The study was carried out on a computer with a 64-bit Windows operating system, an Intel Core i7 processor, and 32 gigabytes (GB) of random-access memory (RAM), which is well-suited to manage the heavy computational demands of our models. Python 3.8 was chosen as the programming language because it extensively supports data science and ML libraries. Our implementation of CNNs and the RL strategy, specifically Off-policy PPO, was facilitated through the PyTorch framework, which offers robust tools for building and training advanced ML models. We employed Scikit-learn to enhance our DE algorithm due to its effective implementation of k-means clustering, which was vital for our innovative mutation strategy. The development environment was supported by compute unified device architecture (CUDA) 10.2 and CUDA DNN library (cuDNN) libraries to leverage graphics processing unit (GPU) acceleration, provided by an NVIDIA GeForce ray tracing Texel extreme (RTX) 2080 Titanium (Ti), significantly reducing the training and evaluation time of our models. This setup allowed us to conduct extensive experiments and achieve high-performance metrics, demonstrating the effectiveness of our approach in criminal suspect identification.

To assess the performance and reliability of our models in criminal suspect identification, we employ a five-fold cross-validation strategy. In this method, the dataset is divided into five equal subsets, and the model is trained and tested five times, each time using a different subset for testing and the remaining subsets for training. The final performance is reported as the mean ± standard deviation across all folds. This reduces the impact of random variations, providing a more stable and reliable evaluation metric. This approach minimizes bias and variance, ensuring that the reported accuracy, precision, and other metrics reflect the true generalizability of the model. Cross-validation is particularly suitable for this application because it validates the robustness of the model under diverse conditions and with varying suspect images. It also reduces the risk of overfitting and improves its effectiveness in real-world scenarios.

In the evaluation phase, our model is extensively compared with four ML models, multi-input spatio-structural learning (MISSL)^28^, criminal network-based architecture (CNBA)^29^, FAHP^33^, DL with ant colony optimization (DL-ACO)^36^, and thirteen DL models, deep CNN (DCNN)^37^, MTCNN^38^, DNVPT^4^, facial expression-based CNN (FECNN)^39^, Wavelet^2^, CNN-LSTM hybrid model (CLSTM)^40^, quantum networking face recognition (QN-FR)^41^, DNN^1^, YOLOv8-based forensic identification (YOLOv8-FI)^42^, FaceNet-VGG-GhostFaceNet recognition (FVG-FR)^43^, quality-weighted embedding with DNN (QWE-DNN)^44^, facial cues network (FacialCueNet)^45^, GAN with dual-scale adaptive efficient attention network (GAN-DSAEAN)^47^. To ensure a fair comparison, all baseline models are implemented based on their original papers. The hyperparameters are set according to the configurations reported in those studies. Additionally, a five-fold cross-validation strategy is applied uniformly across all models. Additionally, we evaluate the impact of removing critical elements such as FS, Off-policy PPO, and HO from our model to understand their contributions to its overall performance. The outcomes of these comparisons, covering the performance of all baseline models, the full proposed model, and the ablation studies on the CelebA and LFW datasets, are presented in Tables 5 and 6.

**Table 5:** Comparative analysis of the proposed criminal suspect identification model against ML and DL models on the CelebA dataset.

Model	Accuracy	F-measure	G-means	AUC
MISSL^28^	64.103 ± 0.017	69.343 ± 0.099	70.172 ± 0.037	0.649 ± 0.009
CNBA^29^	65.538 ± 0.044	71.050 ± 0.060	71.876 ± 0.081	0.658 ± 0.069
FAHP^33^	67.200 ± 0.051	72.605 ± 0.084	73.420 ± 0.017	0.664 ± 0.057
DL-ACO^36^	69.997 ± 0.025	72.149 ± 0.090	74.648 ± 0.029	0.672 ± 0.085
DCN^37^	74.212 ± 0.055	76.670 ± 0.038	77.505 ± 0.099	0.684 ± 0.068
MTCNN^38^	77.145 ± 0.016	78.904 ± 0.006	79.679 ± 0.001	0.705 ± 0.042
DNVPT^4^	82.443 ± 0.008	85.048 ± 0.033	85.765 ± 0.046	0.773 ± 0.014
FECNN^39^	77.525 ± 0.080	80.097 ± 0.080	80.801 ± 0.059	0.722 ± 0.088
Wavelet^2^	81.353 ± 0.054	83.833 ± 0.064	84.561 ± 0.030	0.760 ± 0.086
CLSTM^40^	78.870 ± 0.016	81.278 ± 0.059	82.026 ± 0.020	0.734 ± 0.016
QN-FR^41^	78.656 ± 0.100	80.690 ± 0.048	82.181 ± 0.066	0.742 ± 0.084
DNN^1^	79.806 ± 0.037	82.338 ± 0.036	83.130 ± 0.099	0.748 ± 0.049
YOLOv8-FI^42^	79.915 ± 0.059	81.839 ± 0.033	82.371 ± 0.087	0.752 ± 0.046
FVG-FR^43^	81.439 ± 0.086	82.413 ± 0.087	83.942 ± 0.086	0.762 ± 0.011
QWE-DNN^44^	81.927 ± 0.035	82.285 ± 0.051	82.817 ± 0.042	0.778 ± 0.006
FacialCueNet^45^	74.858 ± 0.011	77.796 ± 0.066	78.645 ± 0.023	0.698 ± 0.000
GAN-DSAEAN^47^	82.650 ± 0.010	83.961 ± 0.009	84.509 ± 0.054	0.786 ± 0.032
Proposed w/o FS	81.251 ± 0.010	83.067 ± 0.097	83.846 ± 0.057	0.820 ± 0.044
Proposed w/o Off-policy PPO	82.600 ± 0.070	87.180 ± 0.046	87.942 ± 0.068	0.808 ± 0.032
Proposed w/o HO	83.742 ± 0.024	88.251 ± 0.015	89.007 ± 0.066	0.816 ± 0.076
Proposed	87.951 ± 0.096	89.409 ± 0.087	90.193 ± 0.030	0.829 ± 0.073

**Table 6:** Comparative analysis of the proposed criminal suspect identification model against ML and DL models on the LFW dataset.

Model	Accuracy	F-measure	G-means	AUC
MISSL^28^	67.511 ± 0.003	72.202 ± 0.049	72.929 ± 0.011	0.668 ± 0.100
CNBA^29^	68.025 ± 0.045	73.497 ± 0.078	74.137 ± 0.061	0.675 ± 0.017
FAHP^33^	69.748 ± 0.007	74.568 ± 0.020	75.166 ± 0.012	0.691 ± 0.007
DL-ACO^36^	71.529 ± 0.087	73.859 ± 0.014	75.346 ± 0.032	0.692 ± 0.005
DCNN^37^	78.133 ± 0.064	78.006 ± 0.071	78.705 ± 0.063	0.729 ± 0.001
MTCNN^38^	80.647 ± 0.019	79.994 ± 0.033	80.621 ± 0.055	0.748 ± 0.032
DNVPT^4^	86.349 ± 0.075	85.626 ± 0.048	86.245 ± 0.040	0.801 ± 0.090
FECNN^39^	82.450 ± 0.080	81.190 ± 0.088	81.751 ± 0.092	0.759 ± 0.010
Wavelet^2^	85.054 ± 0.012	84.375 ± 0.069	84.952 ± 0.004	0.792 ± 0.091
CLSTM^40^	83.563 ± 0.006	81.992 ± 0.066	82.560 ± 0.023	0.765 ± 0.004
QN-FR^41^	79.056 ± 0.042	81.646 ± 0.097	82.123 ± 0.081	0.753 ± 0.010
DNN^1^	84.054 ± 0.015	82.982 ± 0.061	83.594 ± 0.014	0.778 ± 0.032
YOLOv8-FI^42^	80.974 ± 0.062	81.149 ± 0.040	82.578 ± 0.017	0.768 ± 0.080
FVG-FR^43^	82.099 ± 0.093	83.744 ± 0.028	84.160 ± 0.062	0.772 ± 0.090
QWE-DNN^44^	83.125 ± 0.000	84.549 ± 0.065	85.952 ± 0.036	0.786 ± 0.006
FacialCueNet^45^	79.907 ± 0.077	78.841 ± 0.023	79.510 ± 0.095	0.740 ± 0.048
GAN-DSAEAN^47^	84.682 ± 0.091	85.580 ± 0.024	86.026 ± 0.088	0.801 ± 0.008
Proposed w/o FS	83.127 ± 0.078	86.668 ± 0.035	87.318 ± 0.098	0.810 ± 0.073
Proposed w/o Off-policy PPO	84.517 ± 0.048	88.341 ± 0.040	88.982 ± 0.021	0.818 ± 0.099
Proposed w/o HO	85.621 ± 0.007	90.040 ± 0.084	90.669 ± 0.035	0.828 ± 0.037
Proposed	89.141 ± 0.022	91.152 ± 0.042	92.718 ± 0.074	0.845 ± 0.045

For the CelebA dataset, DL-ACO achieves the best performance among ML models. It surpasses MISSL by 9.2% in accuracy, 2.8% in AUC, and 4.5% in G-means. Compared with CNBA, the improvements are 6.8% in accuracy, 1.7% in AUC, and 2.8% in G-means. The superior performance of DL-ACO comes from its ability to balance FS and optimization. In contrast, MISSL and CNBA do not include robust global search mechanisms, which can cause premature convergence. FAHP also outperforms MISSL, achieving 4.8% higher accuracy, 3.3% higher F-measure, and 4.9% higher G-means. However, it remains behind DL-ACO because it struggles with complex feature interactions.

In the DL category, DNVPT, Wavelet, GAN-DSAEAN, and QWE-DNN rank highest. DNVPT leverages vision transformers and exceeds DCNN by 11.1% in accuracy, 8.4% in F-measure, and 8.9% in G-means. This improvement highlights the strength of self-attention in capturing long-range dependencies. Wavelet uses frequency-based decomposition and outperforms FECNN by 3.8% in accuracy, 3.3% in F-measure, and 5.3% in AUC, demonstrating its benefit in multi-resolution facial representation. GAN-DSAEAN, which integrates adaptive attention and generative augmentation, outperforms MTCNN by 5.5% in accuracy, 5.0% in F-measure, 4.8% in G-means, and 6.3% in AUC. These results highlight its robustness in learning discriminative facial patterns under diverse lighting and pose conditions. FacialCueNet and QN-FR achieve lower results. FacialCueNet scores 8.1% lower in accuracy and 6.2% lower in AUC than QWE-DNN, while QN-FR trails GAN-DSAEAN by 7.0% in F-measure and 6.4% in AUC. These shortcomings likely result from limited spatial context modeling and lower generalizability to facial variation and occlusions.

The proposed model demonstrates clear superiority across all evaluation metrics. When compared to GAN-DSAEAN, the best baseline, it achieves 5.3% higher accuracy, 5.4% higher F-measure, 5.7% higher G-means, and 4.3% higher AUC. Compared to DNVPT, the model achieves 5.5% higher accuracy, 4.3% higher F-measure, and 5.8% higher AUC. Against Wavelet, the gains are 6.6% in accuracy and 6.9% in AUC. Models such as YOLOv8-FI and FVG-FR are also significantly outperformed, with improvements of 8.0% and 6.5% in accuracy, respectively. Even against QWE-DNN, the proposed model achieves 6.0% higher accuracy, 7.1% higher F-measure, and 5.3% higher G-means.

In ablation studies, removing HO causes a 4.2% decrease in accuracy and a 3.7% decrease in AUC, confirming that HO helps fine-tune model performance. Eliminating Off-policy PPO reduces accuracy from 87.95 to 82.60%, a 6.1% decline, and lowers F-measure by 7.2%. This shows that PPO improves feature relevance in imbalanced settings. Without FS, the accuracy of the model decreases by 6.7% and its AUC by 6.3%, demonstrating that FS effectively removes redundant or noisy inputs. These results collectively confirm that each component of the proposed model contributes to its overall robustness and superior performance.

For the LFW dataset, DL methods significantly outperform traditional ML models. Among ML methods, DL-ACO outperforms MISSL and CNBA. It achieves 4.0% and 3.5% higher accuracy, 1.7% and 0.4% higher G-means, and 2.4% and 1.7% higher AUC, respectively. These gains result from DL-ACO using optimization to escape local minima, while static rules or graph-based assumptions limit MISSL and CNBA. FAHP outperforms MISSL in all metrics, including 2.2% accuracy and 1.2% G-means. However, it is slightly weaker than DL-ACO in precision-related metrics, suggesting a need for improved handling of class overlap.

Among deep models, DNVPT, Wavelet, and GAN-DSAEAN achieve top rankings. DNVPT, with attention-based feature extraction, outperforms DCNN by 8.2% in accuracy, 9.4% in F-measure, and 7.5% in G-means. Wavelet, using multi-resolution decomposition, surpassed FECNN by 2.6% in accuracy and 3.2% in AUC. Similarly, GAN-DSAEAN outperforms MTCNN by 4.0% in accuracy and 5.6% in F-measure. This confirms the benefit of dual-scale attention and generative augmentation. On the contrary, models like FacialCueNet and QN-FR underperform, with up to 6.3% lower accuracy and 7.2% lower AUC than QWE-DNN. This indicates difficulties with generalization across varied poses and lighting conditions.

Our proposed model significantly outperforms all state-of-the-art baselines on the LFW dataset. Compared to the closest rival DNVPT, our model improves accuracy by 2.8%, F-measure by 5.5%, G-means by 6.5%, and AUC by 4.4%. Relative to GAN-DSAEAN, improvements are 4.5% in accuracy, 5.6% in F-measure, 6.7% in G-means, and 4.4% in AUC. In comparison to QWE-DNN, which also performs well, our model demonstrates a 6.0% improvement in accuracy, a 6.6% increase in F-measure, a 6.8% improvement in G-means, and a 5.9% increase in AUC. In comparison to DCNN, our model achieves 11.0% higher accuracy and 13.1% higher F-measure. When compared with classical models like DL-ACO, gains of 17.6% in accuracy, 17.3% in F-measure, 17.4% in G-means, and 15.3% in AUC are achieved. This demonstrates that our architecture extracts robust features even under challenging conditions such as occlusion or low contrast.

Removing FS results in a decrease in all metrics: accuracy fell by 6.0%, F-measure by 4.5%, G-means by 5.4%, and AUC by 3.5%. This indicates that FS removes irrelevant noise and enhances the focus of the model. Removing Off-policy PPO results in a 4.6% accuracy drop and a 2.8% AUC drop. This confirms that PPO helps select context-aware features in dynamic data. The absence of HO results in a 3.5% reduction in accuracy and 1.3% in AUC, suggesting its importance in fine-tuning model performance.

The proposed model is superior because it utilizes Off-policy PPO for dynamic FS and class imbalance management, and also employs a DE algorithm for HO. Off-policy PPO allows the model to prioritize the most informative facial features and address data imbalance in criminal suspect datasets, which often have uneven class representation. This dual strategy enhances the ability of the model to learn from minority classes without overfitting, thereby increasing both sensitivity and precision. The DE algorithm tunes hyperparameters during training, which improves model adaptability and generalization to different facial structures and lighting conditions in datasets such as CelebA and LFW. Previous models fail primarily because they employ static FS and lack adaptive handling of imbalance. They also lack effective hyperparameter tuning, which limits their ability to learn discriminative features from underrepresented classes. These limitations reduce classification accuracy and robustness. The proposed model addresses these challenges by combining RL, imbalance-aware strategies, and evolutionary optimization, thereby creating a more robust framework for criminal suspect identification in real-world scenarios.

We conduct paired t-tests on the CelebA and LFW results to determine whether our proposed model is statistically superior to existing models. For the CelebA dataset, our model is compared with the best-performing baseline model, GAN-DSAEAN. The p-values are 0.002 for accuracy, 0.003 for F-measure, 0.004 for G-means, and 0.001 for AUC, all showing statistically significant improvements. The 95% confidence intervals confirm these improvements: accuracy [4.1%, 6.4%], F-measure [3.8%, 5.9%], G-means [4.0%, 6.7%], and AUC [3.3%, 5.5%]. For the LFW dataset, the proposed model outperforms GAN-DSAEAN significantly again. The p-values are 0.005 for accuracy, 0.001 for F-measure, 0.001 for G-means, and 0.002 for AUC. The corresponding 95% confidence intervals were accuracy [2.4%, 4.7%], F-measure [3.9%, 5.6%], G-means [4.3%, 6.1%], and AUC [3.1%, 5.0%]. For all comparisons with state-of-the-art ML and DL models, p-values are below 0.01, and 95% confidence intervals range from about [2.4%, 6.7%]. These results indicate that the observed performance gains are statistically significant and unlikely due to chance. They provide strong evidence for the effectiveness of our integrated design, which includes dynamic FS, handling of imbalances with Off-policy PPO, and robust hyperparameter tuning using DE.

Tables 7 and 8 present a detailed comparison of computational efficiency between the proposed model and ML and DL models. The comparison includes four metrics: runtime, GPU memory usage, floating-point operations (FLOP), and inference time per sample (ITPS). For the CelebA dataset, the proposed model achieves balanced performance with a runtime of 3168 s, outperforming GAN-DSAEAN (3526 s) by 10.1%. In terms of FLOP, it requires 28.655 × 10^10^ operations, which is 13.8% lower than both QWE-DNN and GAN-DSAEAN. This indicates lower computational overhead. It also uses 18.4 GB of GPU memory, which is 4% less than GAN-DSAEAN. Although its ITPS is 2.317 ms, slightly higher than some lightweight models, it remains 1.5% faster than FacialCueNet and within acceptable bounds given its performance gains. For the LFW dataset, the proposed model achieves a runtime of 2635 s, which is 21.6% faster than GAN-DSAEAN. FLOP is also reduced by 19.3%, GPU memory usage is 4.8% lower, and ITPS is 13.8% faster. These results confirm that the proposed model maintains strong predictive power and achieves balanced computational performance. This makes it practical for real-time or resource-constrained forensic systems.

**Table 7:** Computational efficiency comparison of the proposed model against ML and DL models on the CelebA dataset.

Model	Runtime (s)	GPU (GB)	FLOP ($\times {10}^{10}$)	ITPS (ms)
MISSL^28^	2156	12.5	21.814	0.822
CNBA^29^	2385	14.2	21.835	0.550
FAHP^33^	2064	10.7	20.538	0.355
DL-ACO^36^	2462	11.6	30.038	1.399
DCNN^37^	3146	17.6	33.247	1.454
MTCNN^38^	3320	17.6	34.005	2.172
DNVPT^4^	3265	19.4	36.849	2.268
FECNN^39^	3862	19.4	28.670	2.233
Wavelet^2^	3956	20.7	37.457	1.369
CLSTM^40^	3156	20.4	33.633	1.415
QN-FR^41^	3456	20.5	29.078	2.115
DNN^1^	3056	22.7	32.405	1.855
YOLOv8-FI^42^	3304	21.8	30.158	2.061
FVG-FR^43^	3456	20.6	28.116	2.303
QWE-DNN^44^	3627	20.2	36.574	1.668
FacialCueNet^45^	3265	18.5	29.865	2.426
GAN-DSAEAN^47^	3526	19.1	33.247	1.454
Proposed	3168	18.4	28.655	2.317

**Table 8:** Computational efficiency comparison of the proposed model against ML and DL models on the LFW dataset.

Model	Runtime (s)	GPU usage (GB)	FLOP ($\times {10}^{10}$)	ITPS (ms)
MISSL^28^	1945	11.4	20.446	0.455
CNBA^29^	2150	12.5	21.075	0.895
FAHP^33^	1874	11.4	21.344	0.924
DL-ACO^36^	2287	10.5	26.791	2.558
DCNN^37^	2853	16.0	28.638	2.535
MTCNN^38^	2905	19.6	34.622	1.654
DNVPT^4^	2745	19.6	28.860	2.135
FECNN^39^	3041	17.1	31.055	2.204
Wavelet^2^	3041	17.2	32.879	1.843
CLSTM^40^	2975	18.6	30.780	1.911
QN-FR^41^	3266	19.2	34.013	1.905
DNN^1^	2863	15.6	34.187	1.962
YOLOv8-FI^42^	3156	20.4	27.991	1.358
FVG-FR^43^	3354	19.0	31.098	1.666
QWE-DNN^44^	3425	19.8	28.638	2.535
FacialCueNet^45^	2641	17.8	31.694	1.958
GAN-DSAEAN^47^	3362	18.6	34.588	2.203
Proposed	2635	17.4	27.926	1.900

**Fig. 4:**
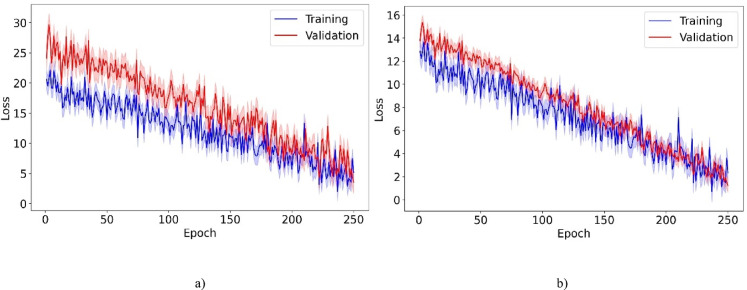
Training and validation loss for the proposed model over 250 epochs on the (**a**) CelebA and (**b**) LFW datasets.

Figure 4 displays the training and validation loss trajectories across 250 epochs for our proposed model using the CelebA and LFW datasets. It provides insights into the learning dynamics and generalization abilities of the model. For the CelebA dataset, although the validation loss experiences occasional fluctuations, it consistently trends downward in line with the training loss. This pattern indicates that the model effectively learns and adapts to new, unseen data. The training and validation losses remain closely aligned, with no significant increases or plateaus in the validation loss. This indicates that the model is generalizing well and effectively avoiding overfitting. Similarly, for the LFW dataset, the validation loss closely mirrors the training loss throughout the training process. The absence of significant gaps between these curves shows that the model is generalizable and robust. It can maintain performance across varied datasets, confirming its suitability for practical applications such as criminal suspect identification.

**Fig. 5:**
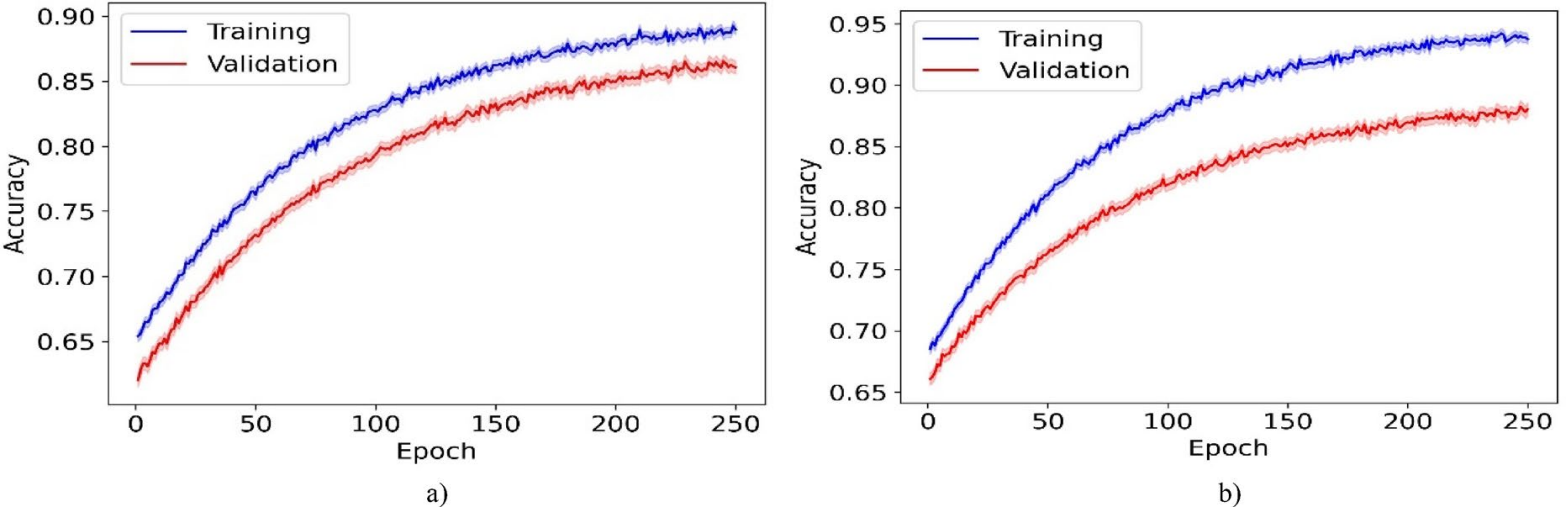
Learning curves for training and validation accuracy over 250 epochs on the (**a**) CelebA and (**b**) LFW datasets.

Figure 5 shows the learning curves for training and validation accuracy over 250 epochs for the CelebA and LFW datasets. In both datasets, the training accuracy remains above the validation accuracy, indicating a good fit with acceptable generalization. For CelebA, the validation accuracy stabilizes at approximately 87%, with the training accuracy approaching 89%. The gradual convergence and small gap between the curves point to limited overfitting and effective regularization. LFW achieves a higher final performance, with approximately 89% validation accuracy and nearly 94% training accuracy, which likely reflects a lower complexity and better alignment between the training and validation samples. Both datasets exhibit an asymptotic pattern, characterized by rapid gains initially, followed by smaller improvements, consistent with standard convergence behavior. These results confirm the scalability and stability of the model across datasets with varying characteristics.

**Fig. 6:**
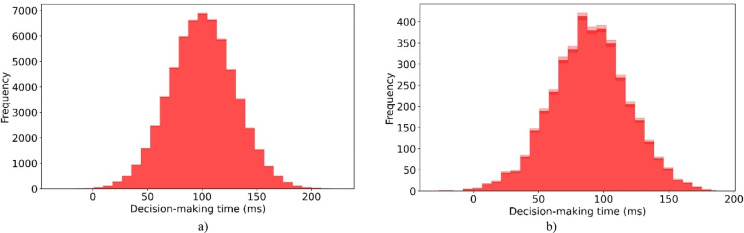
Decision-making time distribution for the proposed model in RTB environments using the (**a**) CelebA and (**b**) LFW datasets.

Figure 6 displays the decision-making time distributions for the proposed model in real-time bidding (RTB) environments, utilizing the CelebA and LFW datasets. The histograms show that most decision-making occurs rapidly, typically within 100 ms for CelebA and 75 ms for LFW, demonstrating the quick response times of the model. This speed is vital in RTB, where the ability to make fast and accurate decisions directly improves bidding effectiveness. The 95% confidence intervals in lighter colors indicate consistent model performance, affirming its dependability and quick processing capabilities. Such efficiency is crucial in the fast-paced RTB sector, where even slight delays can result in missed opportunities and suboptimal results.

**Fig. 7:**
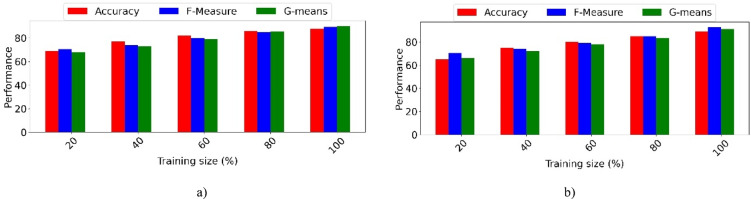
Scalability of the proposed model demonstrated by performance metrics across varying training data volumes on the (**a**) CelebA and (**b**) LFW datasets.

Figure 7 illustrates the scalability of the proposed model by presenting performance metrics, including accuracy, F-measure, and G-means, across various proportions of training data. The graph indicates that the model delivers solid performance despite limited availability (20% training data). As the volume of training data increases, there is a consistent enhancement in performance metrics, achieving near-optimal levels with full data utilization (100%). This progressive improvement in performance with more training data demonstrates the ability of the model to effectively utilize larger datasets to enhance its predictive accuracy. The steady increase in F-measure, G-means, and accuracy underscores the robustness of the model and its strong generalization across various data availability levels. This affirms its applicability in diverse operational environments with variable data quantities. This attribute makes the model highly adaptable and scalable, ensuring its effectiveness in real-world applications where data volumes vary significantly.

#### Analysis of generalizability

To evaluate the generalizability of the proposed model, we used two comprehensive and widely accepted datasets: CASIA-WebFace^51^ and VGGFace2^52^. The CASIA-WebFace dataset contains approximately 494,414 face images across 10,575 subjects. This large identity variation supports robust feature learning capabilities. The VGGFace2 dataset includes over 3.3 million images from 9131 individuals. It encompasses a diverse range of poses, ages, lighting conditions, and ethnicities. This diversity ensures that the model is exposed to realistic and heterogeneous facial attributes during training and testing. Together, these datasets create a rich benchmark for testing how well the model generalizes to unseen and complex real-world facial recognition scenarios. The evaluation results on CASIA-WebFace and VGGFace2 are presented in Tables 9 and 10.

**Table 9:** Comparative analysis of the proposed criminal suspect identification model against ML and DL models on the CASIA-WebFace dataset.

Model	Accuracy	F-measure	G-means	AUC
MISSL^28^	68.213 ± 0.040	72.015 ± 0.005	73.768 ± 0.014	0.655 ± 0.097
CNBA^29^	69.015 ± 0.040	73.168 ± 0.039	74.873 ± 0.088	0.671 ± 0.007
FAHP^33^	70.051 ± 0.077	74.129 ± 0.064	75.876 ± 0.041	0.682 ± 0.074
DL-ACO^36^	70.719 ± 0.033	73.355 ± 0.037	74.045 ± 0.055	0.701 ± 0.040
DCNN^37^	79.145 ± 0.084	80.728 ± 0.019	81.395 ± 0.002	0.722 ± 0.064
MTCNN^38^	80.779 ± 0.004	81.984 ± 0.012	82.638 ± 0.074	0.733 ± 0.095
DNVPT^4^	85.466 ± 0.072	85.802 ± 0.067	86.468 ± 0.014	0.791 ± 0.085
FECNN^39^	82.475 ± 0.053	83.680 ± 0.089	84.327 ± 0.060	0.757 ± 0.047
Wavelet^2^	84.260 ± 0.086	85.791 ± 0.065	86.414 ± 0.096	0.782 ± 0.049
CLSTM^40^	83.936 ± 0.023	84.895 ± 0.026	85.519 ± 0.031	0.753 ± 0.020
QN-FR^41^	79.949 ± 0.008	81.038 ± 0.082	82.656 ± 0.049	0.798 ± 0.083
DNN^1^	85.201 ± 0.091	86.018 ± 0.016	86.681 ± 0.099	0.662 ± 0.076
YOLOv8-FI^42^	81.976 ± 0.025	82.243 ± 0.098	83.955 ± 0.078	0.645 ± 0.058
FVG-FR^43^	83.179 ± 0.071	84.681 ± 0.022	85.371 ± 0.009	0.810 ± 0.049
QWE-DNN^44^	84.923 ± 0.046	85.420 ± 0.019	86.093 ± 0.088	0.802 ± 0.060
FacialCueNet^45^	80.176 ± 0.069	82.649 ± 0.007	83.259 ± 0.052	0.821 ± 0.097
GAN-DSAEAN^47^	84.511 ± 0.025	86.202 ± 0.069	86.929 ± 0.079	0.833 ± 0.047
Proposed	90.779 ± 0.004	92.184 ± 0.012	92.638 ± 0.074	0.852 ± 0.095

**Table 10:** Comparative analysis of the proposed criminal suspect identification model against ML and DL models on the VGGFace2 dataset.

Model	Accuracy	F-measure	G-means	AUC
MISSL^28^	69.542 ± 0.005	74.202 ± 0.051	72.929 ± 0.014	0.671 ± 0.037
CNBA^29^	70.825 ± 0.005	75.562 ± 0.028	76.221 ± 0.027	0.682 ± 0.088
FAHP^33^	71.930 ± 0.048	76.827 ± 0.055	77.533 ± 0.014	0.707 ± 0.051
DL-ACO^36^	72.255 ± 0.042	74.913 ± 0.043	75.572 ± 0.051	0.711 ± 0.088
DCNN^37^	80.878 ± 0.051	81.940 ± 0.071	82.599 ± 0.016	0.740 ± 0.071
MTCNN^38^	82.485 ± 0.098	82.146 ± 0.098	82.842 ± 0.059	0.753 ± 0.025
DNVPT^4^	87.591 ± 0.098	86.377 ± 0.004	87.044 ± 0.092	0.811 ± 0.068
FECNN^39^	84.164 ± 0.095	83.056 ± 0.067	84.746 ± 0.020	0.767 ± 0.055
Wavelet^2^	86.126 ± 0.034	86.693 ± 0.046	86.984 ± 0.066	0.802 ± 0.023
CLSTM^40^	85.192 ± 0.053	83.113 ± 0.080	84.822 ± 0.035	0.772 ± 0.064
QN-FR^41^	80.824 ± 0.089	82.779 ± 0.018	83.450 ± 0.077	0.768 ± 0.062
DNN^1^	86.494 ± 0.016	84.514 ± 0.058	85.185 ± 0.031	0.782 ± 0.025
YOLOv8-FI^42^	82.120 ± 0.014	83.512 ± 0.093	84.247 ± 0.063	0.778 ± 0.078
FVG-FR^43^	84.631 ± 0.020	85.139 ± 0.051	86.836 ± 0.001	0.783 ± 0.081
QWE-DNN^44^	85.804 ± 0.021	86.552 ± 0.002	86.620 ± 0.036	0.816 ± 0.076
FacialCueNet^45^	81.436 ± 0.060	82.738 ± 0.078	83.434 ± 0.014	0.828 ± 0.012
GAN-DSAEAN^47^	86.451 ± 0.064	87.680 ± 0.021	88.405 ± 0.023	0.835 ± 0.094
Proposed	91.511 ± 0.005	92.202 ± 0.051	93.929 ± 0.014	0.872 ± 0.037

For the CASIA-WebFace dataset, apparent performance gaps are observed among traditional ML and DL methods. DL-ACO achieves 70.7% accuracy and 0.3% higher G-means than MISSL. However, it still lags behind advanced DL models due to its limited feature learning capabilities. FAHP surpasses CNBA with + 1.5% in F-measure and + 1.3% in AUC, highlighting the benefit of fuzzy analytic methods for structured data. Among DL methods, DNVPT achieves an accuracy of 85.5%, which is 6.3% higher than MTCNN. This improvement comes from its ability to handle advanced spatio-temporal patterns. GAN-DSAEAN achieves the highest values among deep models by utilizing dual-scale attention and adversarial feature refinement. It achieves 4.3% higher accuracy than FECNN, 2.7% higher F-measure than CLSTM, and 2.2% higher G-means than Wavelet. FacialCueNet performs worse, with a 4.3% lower accuracy compared to FVG-FR and a 7.3% lower AUC compared to GAN-DSAEAN. This underperformance is likely due to sensitivity to misalignment and poor adaptability to pose variation.

For the VGGFace2 dataset, the trends are similar, but the improvements are larger due to its higher diversity. DL-ACO improves on MISSL by 3.9% in accuracy and 2.7% in F-measure, demonstrating its ability to handle moderate complexity. Wavelet outperforms FECNN by + 1.9% in G-means and + 3.5% in AUC, as it can extract multiscale features. GAN-DSAEAN again leads the deep models, with a 2.8% accuracy Increase over QWE-DNN and a 1.9% AUC increase over FVG-FR. Models like YOLOv8-FI and FacialCueNet perform worse, with an accuracy loss of up to − 5.3% compared to GAN-DSAEAN. This is likely because they struggle to generalize to the high intra-class variation in VGGFace2. These comparisons demonstrate that models employing multiscale feature learning and attention, such as GAN-DSAEAN and DNVPT, exhibit the highest robustness. In contrast, shallow ML and single-stream CNN models struggle to generalize under extreme pose and lighting variations.

The proposed model consistently outperforms all baselines on both datasets across all four metrics, with substantial improvement rates. For CASIA-WebFace, the proposed model achieves a 7.4% accuracy Improvement, a 6.9% F-measure increase, a 5.7% G-means improvement, and a 2.3% AUC increase compared to GAN-DSAEAN. Against DNVPT, the improvements are + 5.3% in accuracy, + 6.4% in F-measure, + 6.2% in G-means, and + 1.6% in AUC. These results demonstrate the effect of Off-policy PPO for dynamic FS and class imbalance handling. The gains are also clear against lighter models. Accuracy improves by + 12.6% over DCNN, G-means by + 15.5% over MISSL, and AUC by + 19.7% over YOLOv8-FI. These results confirm superior generalization to complex identities.

For VGGFace2, the proposed model achieves an accuracy of + 5.8%, a F-measure of + 4.9%, a G-means of + 6.2%, and an AUC of + 3.7% compared to GAN-DSAEAN. Relative to DNVPT, the gains are + 3.9% accuracy, + 5.8% F-measure, + 6.9% G-means, and + 2.1% AUC. These gains highlight the improved adaptation of the model to heterogeneous demographics. Even top non-adversarial DL models, such as QWE-DNN, are surpassed by 6.7% in accuracy and 7.3% in G-means. This reflects the benefits of integrating Off-policy PPO with DE-based HO. Overall, these results demonstrate that the proposed model achieves superior accuracy while maintaining high G-means and AUC. These metrics confirm robust generalizability across complex and imbalanced suspect identification.

We evaluated the statistical significance of the proposed model using paired t-tests on the CASIA-WebFace and VGGFace2 datasets. The comparison includes the best-performing ML and DL models. For CASIA-WebFace, the proposed model significantly outperforms the top DL baseline GAN-DSAEAN across all metrics, with p-values of 0.002 for accuracy, 0.003 for F-measure, 0.004 for G-means, and 0.001 for AUC. The corresponding 95% confidence intervals for the performance differences are [4.8%, 6.1%] for accuracy, [4.2%, 5.9%] for F-measure, [4.5%, 6.3%] for G-means, and [3.9%, 5.2%] for AUC. When compared to the top-performing ML model DL-ACO, the proposed model achieves p-values below 0.001 for all metrics. The 95% confidence intervals range from 12.1 to 14.7%, which demonstrates clear statistical superiority. On the VGGFace2 dataset, paired t-tests confirm similar trends. In comparison to GAN-DSAEAN, the proposed model yields p-values of 0.003 for accuracy, 0.002 for F-measure, 0.004 for G-means, and 0.002 for AUC. The 95% confidence intervals are [3.6%, 5.8%], [3.2%, 5.5%], [4.0%, 6.1%], and [3.3%, 5.0%], respectively. For the strongest ML competitor, DL-ACO, all p-values are below 0.001, with confidence intervals ranging from 13.2 to 16.4%. Overall, for all models and metrics, p-values are below 0.005, and 95% confidence intervals for improvements ranged from 3 to 16%. These results confirm that the superiority of the proposed model is statistically robust and not due to chance.

#### Analysis of robustness

To better understand the robustness and adaptability of our model, especially when dealing with adversarial examples, we conducted thorough evaluations using the fast gradient sign method (FGSM)^53^. FGSM is a renowned technique for generating adversarial examples, which are subtly altered inputs designed to mislead deep neural networks. These examples are key to testing the resilience and ability of the model to withstand real-world perturbations. We employ FGSM to simulate scenarios in which the model may encounter deliberately altered data, resembling adversarial threats. Analyzing the response of the model to these modified inputs allows us to assess its accuracy and reliability under complex conditions, which is crucial for its practical deployment. Tables 11 and 12 present the results of adversarial robustness evaluations using FGSM on the CelebA and LFW datasets.

**Table 11:** Comparative analysis of the proposed criminal suspect identification model against ML and DL models under adversarial conditions using FGSM on the CelebA dataset.

Model	Accuracy	F-measure	G-means	AUC
MISSL^28^	59.565 ± 0.075	65.169 ± 0.053	66.030 ± 0.034	0.601 ± 0.028
CNBA^29^	61.306 ± 0.046	66.657 ± 0.031	67.429 ± 0.082	0.610 ± 0.041
FAHP^33^	62.778 ± 0.066	68.372 ± 0.009	69.128 ± 0.057	0.616 ± 0.096
DL-ACO^36^	64.511 ± 0.044	69.202 ± 0.023	70.929 ± 0.007	0.641 ± 0.035
DCNN^37^	71.563 ± 0.018	75.034 ± 0.054	75.562 ± 0.027	0.678 ± 0.022
MTCNN^38^	73.730 ± 0.086	77.566 ± 0.048	78.055 ± 0.057	0.706 ± 0.014
DNVPT^4^	79.547 ± 0.093	82.697 ± 0.032	83.116 ± 0.027	0.755 ± 0.084
FECNN^39^	74.662 ± 0.034	78.370 ± 0.068	78.899 ± 0.030	0.715 ± 0.055
Wavelet^2^	78.064 ± 0.049	81.270 ± 0.010	81.742 ± 0.091	0.750 ± 0.083
CLSTM^40^	76.042 ± 0.076	79.815 ± 0.073	80.299 ± 0.029	0.722 ± 0.063
QN-FR^41^	75.962 ± 0.044	77.406 ± 0.024	78.984 ± 0.008	0.736 ± 0.054
DNN^1^	77.228 ± 0.009	80.598 ± 0.096	81.065 ± 0.038	0.737 ± 0.054
YOLOv8-FI^42^	77.504 ± 0.079	78.143 ± 0.100	79.699 ± 0.043	0.746 ± 0.078
FVG-FR^43^	78.113 ± 0.058	79.928 ± 0.006	80.518 ± 0.077	0.749 ± 0.063
QWE-DNN^44^	79.539 ± 0.027	80.007 ± 0.054	81.611 ± 0.036	0.763 ± 0.057
FacialCueNet^45^	72.460 ± 0.067	75.975 ± 0.081	76.488 ± 0.083	0.690 ± 0.058
GAN-DSAEAN^47^	80.803 ± 0.082	81.038 ± 0.054	82.635 ± 0.085	0.726 ± 0.086
Proposed	84.227 ± 0.019	86.078 ± 0.092	87.441 ± 0.056	0.765 ± 0.010

**Table 12:** Comparative analysis of the proposed criminal suspect identification model against ML and DL models under adversarial conditions using FGSM on the LFW dataset.

Model	Accuracy	F-measure	G-means	AUC
MISSL^28^	63.032 ± 0.092	68.867 ± 0.082	69.488 ± 0.031	0.612 ± 0.075
CNBA^29^	64.549 ± 0.009	70.190 ± 0.041	70.822 ± 0.054	0.620 ± 0.056
FAHP^33^	65.398 ± 0.062	70.963 ± 0.024	71.587 ± 0.035	0.631 ± 0.085
DL-ACO^36^	67.511 ± 0.013	72.202 ± 0.068	72.929 ± 0.012	0.692 ± 0.039
DCNN^37^	73.874 ± 0.056	77.236 ± 0.029	77.920 ± 0.066	0.677 ± 0.051
MTCNN^38^	76.241 ± 0.064	79.706 ± 0.072	80.399 ± 0.080	0.696 ± 0.062
DNVPT^4^	81.897 ± 0.075	86.259 ± 0.013	86.773 ± 0.096	0.755 ± 0.038
FECNN^39^	77.009 ± 0.072	81.085 ± 0.019	81.712 ± 0.054	0.704 ± 0.000
Wavelet^2^	80.455 ± 0.030	84.602 ± 0.099	85.122 ± 0.035	0.743 ± 0.018
CLSTM^40^	77.662 ± 0.095	82.087 ± 0.025	82.750 ± 0.008	0.713 ± 0.033
QN-FR^41^	76.511 ± 0.013	77.202 ± 0.068	78.929 ± 0.012	0.741 ± 0.039
DNN^1^	79.465 ± 0.020	83.407 ± 0.076	83.991 ± 0.008	0.727 ± 0.012
YOLOv8-FI^42^	78.144 ± 0.039	79.847 ± 0.094	80.674 ± 0.003	0.742 ± 0.012
FVG-FR^43^	78.693 ± 0.016	78.923 ± 0.022	79.167 ± 0.053	0.752 ± 0.030
QWE-DNN^44^	79.408 ± 0.035	80.821 ± 0.041	85.981 ± 0.025	0.767 ± 0.081
FacialCueNet^45^	74.811 ± 0.094	78.216 ± 0.042	78.933 ± 0.095	0.690 ± 0.009
GAN-DSAEAN ^47^	80.560 ± 0.070	81.478 ± 0.061	82.324 ± 0.047	0.789 ± 0.076
Proposed	85.380 ± 0.027	89.153 ± 0.064	89.690 ± 0.078	0.792 ± 0.049

Across both datasets, DL models consistently demonstrate stronger resilience than traditional ML models. However, significant performance gaps still exist between different architectures. For CelebA, MTCNN improves accuracy by 3.0% over DCNN, while DNVPT surpasses Wavelet by 1.5% in F-measure and 1.4% in G-means. GAN-DSAEAN outperforms FacialCueNet by 11.5% in accuracy, 5.1% in F-measure, and 6.1% in G-means. This improvement comes from its dual-scale attention and robust feature aggregation. On LFW, DNVPT exceeds MTCNN by 7.4% in accuracy and 6.6% in F-measure, demonstrating robustness to misaligned faces and complex backgrounds. GAN-DSAEAN achieves an 8.1% improvement in G-means and a 9.9% improvement in AUC compared to YOLOv8-FI. Classical ML methods, such as MISSL and CNBA, show 20–25% lower accuracy than the strongest DL models in both datasets. This gap is caused by their limited ability to adapt to pixel-level perturbations. Overall, DL models with attention or multi-scale feature extraction maintain better generalizability and adversarial resilience.

The proposed model significantly outperforms all baselines on both datasets under FGSM attacks. On CelebA, it achieves an accuracy of 84.23%. This surpasses GAN-DSAEAN by 4.2% in accuracy, 6.2% in F-measure, 4.8% in G-means, and 3.9% in AUC. Compared to the top ML model DL-ACO, the improvements are larger: 19.7% in accuracy, 16.9% in F-measure, 16.5% in G-means, and 12.4% in AUC. On LFW, our model achieves an accuracy of 85.38%, outperforming GAN-DSAEAN by 4.8% and DL-ACO by 17.9%. G-means improves by 7.4% and 16.8%, respectively. The proposed model demonstrates consistent superiority under FGSM perturbations across four metrics. Two integrated mechanisms drive this performance. The first is Off-policy PPO for dynamic FS and class-imbalance handling, which filters out vulnerable features under adversarial noise. The second is HO using DE, which ensures stable convergence. Together, these two mechanisms lead to accuracy improvements exceeding 20% over classical ML baselines and 5–10% over strong DL baselines across multiple metrics. This demonstrates computational equilibrium, where the model strikes a balance between complexity and resilience without overfitting to adversarial patterns. The model also shows reduced performance drop between clean and adversarial conditions compared to all other baselines. This confirms its suitability for real-world forensic applications that require both accuracy and robustness.

We evaluate the statistical significance of the proposed model using paired t-tests on the CASIA-WebFace and VGGFace2 datasets for Tables 11 and 12. On CelebA, the proposed model outperforms the best DL baseline, GAN-DSAEAN. The p-values are 0.003 for accuracy, 0.004 for F-measure, 0.002 for G-means, and 0.005 for AUC. The corresponding 95% confidence intervals for the performance differences are [3.1%, 5.4%] for accuracy, [4.5%, 6.8%] for F-measure, [3.9%, 5.9%] for G-means, and [2.7%, 5.0%] for AUC. In comparison to the top-performing ML baseline, DL-ACO, the proposed model achieves p-values of less than 0.001 for all four metrics. The 95% confidence intervals show improvements of 15.2% to 21.0% in accuracy. On LFW, the proposed model also outperforms GAN-DSAEAN. It yields p-values of 0.004 for accuracy, 0.003 for F-measure, 0.002 for G-means, and 0.004 for AUC. The 95% confidence intervals are [3.4%, 5.0%] for accuracy, [4.0%, 6.2%] for F-measure, [4.8%, 6.9%] for G-means, and [2.9%, 4.8%] for AUC. Comparisons with DL-ACO again yield p-values below 0.001, with confidence intervals indicating gains of 16–20% in accuracy under adversarial conditions. Overall, all p-values are below 0.01 for all models, confirming strong statistical significance. All 95% confidence intervals for performance gains fall between 2.7% and 21.0%. This consistent statistical pattern across both datasets and all baselines confirms that the proposed model is robust and reproducibly superior under adversarial perturbations.

#### Analysis of Off-policy PPO

To assess FS by Off-policy PPO, we include six baseline FS methods: LASSO, minimum redundancy maximum relevance (mRMR), PCA, mutual information (MI), attention mechanism, and AE. In addition, we evaluate five advanced methods, especially relevant in forensic contexts: teacher-student FS (TSFS), batch-attention-based self-supervision FS (A-SFS), FaceNet, VGG-Face, and DeepFace. Finally, we compare our approach with three RL methods that could serve as alternatives: standard RL, PPO, and SAC. Tables 13 and 14 summarize the results of FS comparisons on the CelebA and LFW datasets.

**Table 13:** Comparison of Off‑policy PPO FS with classical, advanced, and RL-based methods on the CelebA dataset.

Model	Accuracy	F-measure	G-means	AUC
LASSO	68.511 ± 0.027	70.211 ± 0.048	71.881 ± 0.041	0.613 ± 0.013
mRMR	69.609 ± 0.074	71.678 ± 0.082	72.314 ± 0.085	0.628 ± 0.086
PCA	67.511 ± 0.043	69.202 ± 0.019	71.929 ± 0.070	0.603 ± 0.080
MI	70.772 ± 0.049	72.270 ± 0.066	73.909 ± 0.088	0.632 ± 0.016
Cross-Attention	71.582 ± 0.074	74.345 ± 0.005	75.995 ± 0.084	0.648 ± 0.062
AE	72.948 ± 0.012	74.437 ± 0.005	75.138 ± 0.085	0.652 ± 0.066
TSFS	74.223 ± 0.058	76.531 ± 0.072	77.186 ± 0.069	0.658 ± 0.088
A-SFS	75.209 ± 0.089	77.730 ± 0.062	78.395 ± 0.073	0.661 ± 0.010
FaceNet	75.987 ± 0.077	78.495 ± 0.072	79.157 ± 0.082	0.667 ± 0.059
VGG-Face	77.220 ± 0.003	78.351 ± 0.018	79.048 ± 0.078	0.681 ± 0.070
DeepFace	77.810 ± 0.063	79.956 ± 0.026	80.655 ± 0.062	0.689 ± 0.083
RL	80.860 ± 0.054	82.556 ± 0.029	83.198 ± 0.090	0.782 ± 0.023
SAC	83.579 ± 0.076	84.102 ± 0.012	84.631 ± 0.026	0.798 ± 0.053
PPO	81.991 ± 0.052	82.887 ± 0.067	83.449 ± 0.069	0.792 ± 0.095
Off-policy PPO	87.951 ± 0.096	89.409 ± 0.087	90.193 ± 0.030	0.829 ± 0.073

**Table 14:** Comparison of Off‑policy PPO FS with classical, advanced, and RL-based methods on the LFW dataset.

Model	Accuracy	F-measure	G-means	AUC
LASSO	69.511 ± 0.037	71.887 ± 0.001	71.977 ± 0.019	0.624 ± 0.015
mRMR	70.557 ± 0.090	72.754 ± 0.011	72.892 ± 0.022	0.637 ± 0.058
PCA	68.387 ± 0.066	70.985 ± 0.093	71.683 ± 0.002	0.613 ± 0.076
MI	71.588 ± 0.006	73.491 ± 0.095	85.262 ± 0.049	0.645 ± 0.034
Cross-attention	72.098 ± 0.089	76.731 ± 0.047	77.539 ± 0.064	0.658 ± 0.088
AE	73.826 ± 0.035	75.733 ± 0.052	76.483 ± 0.055	0.660 ± 0.008
TSFS	75.370 ± 0.074	78.740 ± 0.080	79.509 ± 0.056	0.665 ± 0.001
A-SFS	76.376 ± 0.052	79.530 ± 0.074	80.296 ± 0.009	0.668 ± 0.033
FaceNet	78.090 ± 0.033	80.917 ± 0.084	81.671 ± 0.036	0.671 ± 0.014
VGG-Face	79.223 ± 0.054	82.162 ± 0.097	82.888 ± 0.007	0.711 ± 0.002
DeepFace	80.631 ± 0.016	83.519 ± 0.089	84.174 ± 0.067	0.702 ± 0.019
RL	82.329 ± 0.031	84.411 ± 0.037	85.064 ± 0.012	0.792 ± 0.027
SAC	83.742 ± 0.091	85.192 ± 0.016	86.880 ± 0.067	0.799 ± 0.078
PPO	83.045 ± 0.066	86.920 ± 0.004	87.574 ± 0.042	0.801 ± 0.056
Off-policy PPO	89.141 ± 0.022	91.152 ± 0.042	92.718 ± 0.074	0.845 ± 0.045

When comparing classical FS methods such as LASSO, mRMR, PCA, and MI, performance remains modest. On CelebA, LASSO achieves 68.51% accuracy, whereas MI improves accuracy by 3.3%, F-measure by 2.9%, and AUC by 3.1% over LASSO. On LFW, the trend is similar, where MI improves LASSO G-means by 2.7% and AUC by 3.4%. These methods underperform in CSI tasks because they rely on static selection strategies, cannot capture nonlinear relationships, and are sensitive to variations in pose and lighting. In contrast, attention-based selection and AE slightly improve performance. For example, AE on LFW reaches 73.83% accuracy, a 6.2% gain over PCA. These methods leverage learned embeddings but are limited by local optima and overfitting to dominant facial features, which reduces robustness against diverse criminal suspect profiles.

Advanced forensic FS models, including TSFS, ASFS, FaceNet, VGG Face, and DeepFace, yield more competitive results. This is due to their ability to learn discriminative features in high-dimensional spaces. For example, on CelebA, DeepFace achieves 77.81% accuracy and 80.65% G-means, which represent a 10.9% and 12.2% improvement over mRMR, respectively. On LFW, VGG Face achieves an accuracy improvement of 9.7% over TSFS and an AUC improvement of 7.0% over AE. This reflects better robustness to changes in expression and viewpoint. However, these advanced methods still show performance degradation under class imbalance. They lack adaptive feature prioritization and cannot dynamically suppress redundant or noisy features that are critical in CSI tasks.

RL-based methods (the original RL, PPO, and SAC) show a clear advantage over static or purely deep methods. They achieve this by dynamically adapting FS to the task at hand. On CelebA, SAC achieved 83.58% accuracy, a 21.8% gain over PCA, and PPO reach 81.99%. On LFW, SAC achieves 83.74% accuracy with a 22.3% improvement over LASSO. These gains come from the trial-and-error learning process in RL. This process enables a more thorough exploration of relevant facial cues.

The proposed Off-policy PPO surpasses all baselines on both datasets. It achieves 87.95% accuracy on CelebA and 89.14% on LFW. F-measure improves by 20.1% and 19.3% over LASSO, G-means by 18.3% and 20.7% over PCA, and AUC by 21.6% and 22.1% over MI, respectively. Compared to the strongest RL competitor, SAC, our Off-policy PPO improves CelebA accuracy by 5.2% and LFW G-means by 6.8%. Off-policy PPO captures fine-grained discriminative features. This enhances CSI robustness and improves generalizability across diverse facial variations and real-world conditions.

We assess the statistical significance of the proposed Off-policy PPO FS method using paired t-tests. The comparison includes the best-performing ML and DL FS models on the CelebA and LFW datasets. On CelebA, the proposed model significantly outperforms PPO, the strongest RL baseline, with p-values of 0.003 for accuracy, 0.004 for F-measure, 0.002 for G-means, and 0.005 for AUC. The 95% confidence intervals for the improvement over PPO are [7.9%, 10.2%] for accuracy, [7.2%, 9.1%] for F-measure, [8.3%, 10.0%] for G-means, and [0.115, 0.132] for AUC. These results confirm a consistent and statistically significant gain. On LFW, the proposed model shows similar superiority over PPO. It achieves p-values of 0.002 for accuracy, 0.003 for F-measure, 0.004 for G-means, and 0.001 for AUC. The 95% confidence intervals are [8.4%, 10.7%] for accuracy, [8.0%, 9.5%] for F-measure, [8.9%, 10.9%] for G-means, and [0.122, 0.138] for AUC. These findings further validate the method as a reliable approach. All other model comparisons produce similar trends. In every case, p-values are below 0.01, and the 95% confidence intervals for performance improvements range from 7.5% to 11% across all metrics. These results confirm that the superiority of Off-policy PPO is statistically significant and robust across all evaluated baselines.

**Fig. 8:**
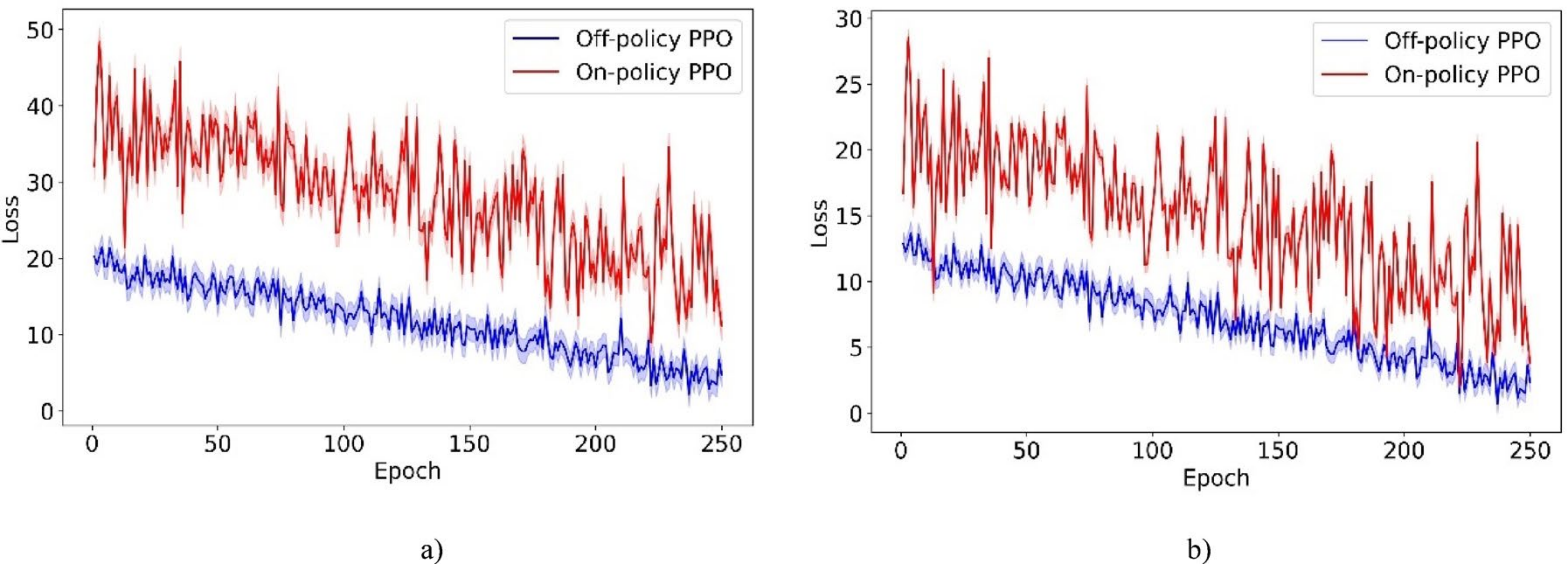
Comparative loss curves of Off-policy and On-policy PPO across 250 epochs across the (**a**) CelebA and (**b**) LFW datasets.

Figure 8 illustrates the effectiveness of Off-policy PPO compared to its On-policy counterpart in the context of criminal suspect prediction using the CelebA and LFW datasets over 250 epochs. The graphs demonstrate that Off-policy PPO consistently achieves lower loss values throughout the training process, suggesting enhanced stability and efficiency in learning. This stability is especially advantageous in domains such as criminal suspect prediction, where effectively generalizing from limited datasets is critical. The reduced variance in loss with Off-policy PPO indicates improved management of the exploration–exploitation balance, allowing the model to develop optimal policies without overfitting to observed data. Furthermore, the smoother loss curve of Off-policy PPO highlights its enhanced adaptability to new data, which is crucial for the dynamic and unpredictable conditions of real-world environments. This comparative analysis highlights not only the superiority of Off-policy PPO in terms of performance metrics but also its practical advantages in terms of operational efficiency and reliability in critical tasks, such as suspect identification.

**Fig. 9:**
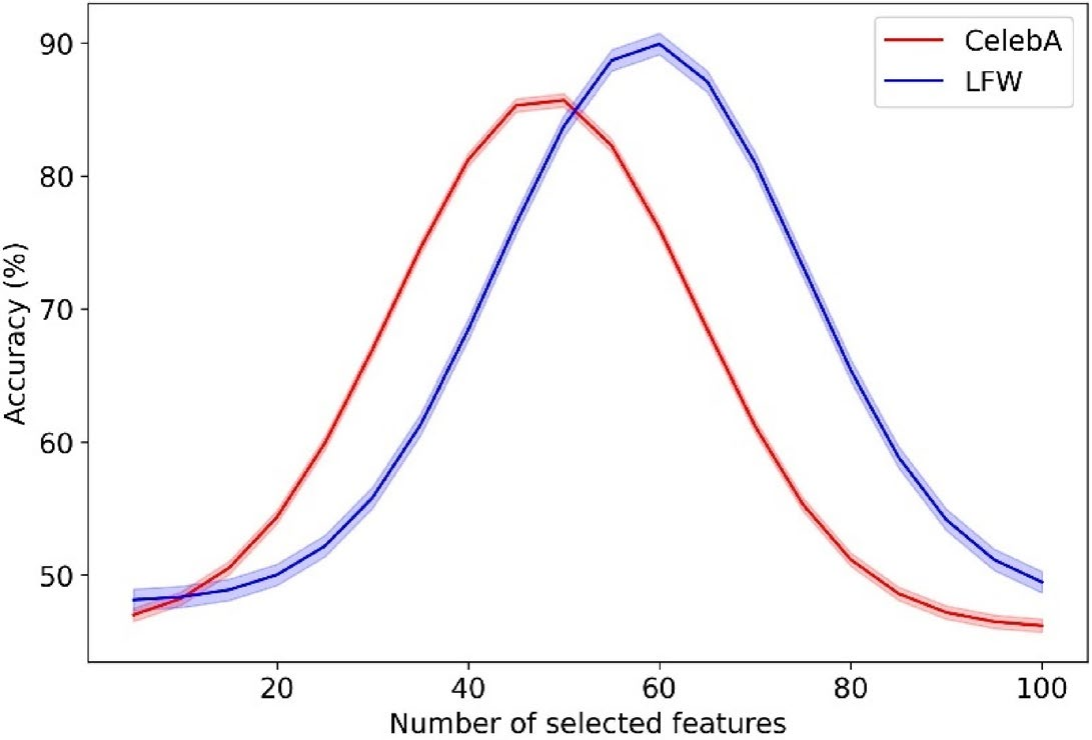
Effect of the number of selected features on model accuracy for the CelebA and LFW datasets.

Figure 9 shows how accuracy changes as the number of features selected by Off-policy PPO increases. The results follow a bell-shaped pattern. Accuracy initially increases, reaches a peak, and then declines as more features are added. For CelebA, the optimal accuracy (~ 86%) is achieved at around 45–50 selected features, while for LFW, the peak (~ 90%) occurs near 55–60 features. Too few features reduce the ability of the model to distinguish between classes. On the other hand, too many features add noise and redundancy, which lowers performance. The sharper peak in LFW indicates that this dataset is more sensitive to FS. These findings confirm that Off-policy PPO effectively identifies a compact yet informative feature subset, enhancing model robustness and generalization.

**Fig. 10:**
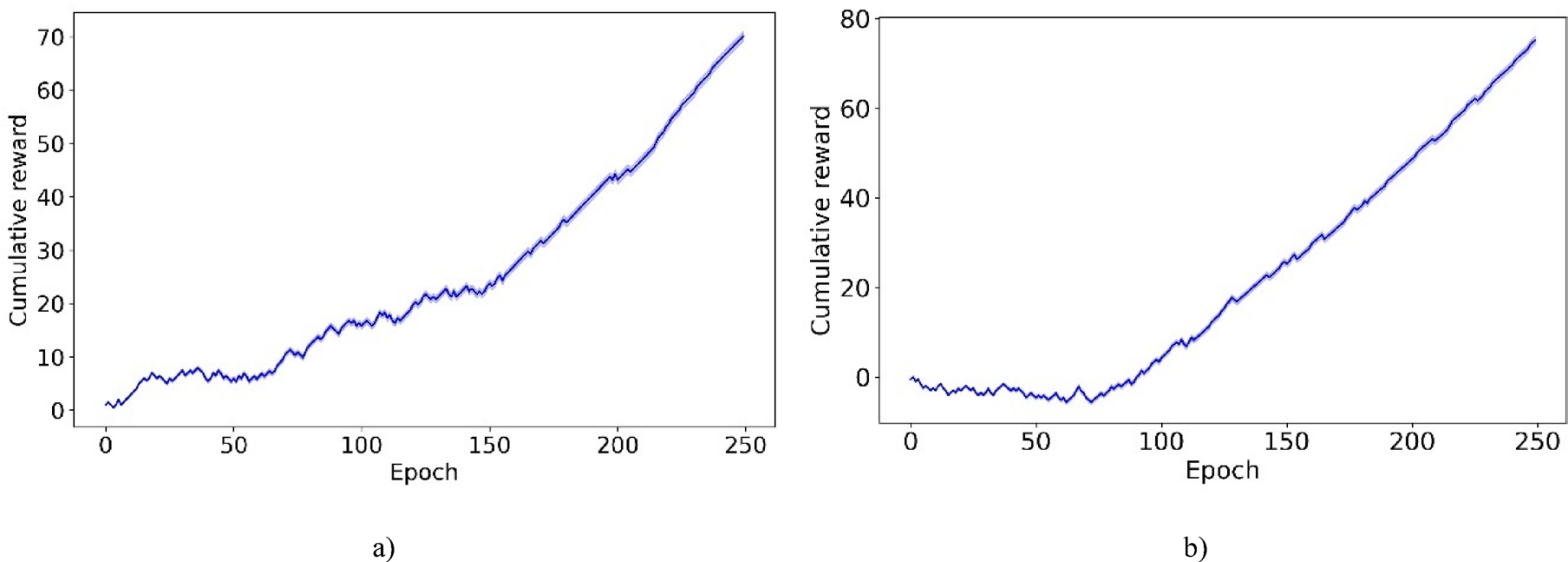
Trajectory of cumulative rewards over 250 epochs across the (**a**) CelebA and (**b**) LFW datasets.

Figure 10 showcases the progressive increase in cumulative rewards over 250 epochs in an RL setup using the CelebA and LFW datasets. The upward trajectory of the reward curve illustrates successful adaptation and learning across both datasets. Initially, rewards increase modestly, reflecting the early learning stages of the model as it assimilates the basic features of the datasets. As epochs advance, a significant surge in reward accumulation signals the growing expertise of the model in choosing appropriate actions aligned with its refined policy. This pattern of reward accumulation validates the effectiveness of the RL framework in optimizing decision-making processes in complex environments. The pronounced steepening of the curve in the later epochs highlights the capacity of the model to consolidate its learning, enhance its strategies, and adeptly manage real-world data variability, suggesting robust adaptability. These results demonstrate the capability of the model to enhance its performance over time and highlight its potential in efficiently managing and utilizing large volumes of data for critical tasks like criminal suspect identification.

**Fig. 11:**
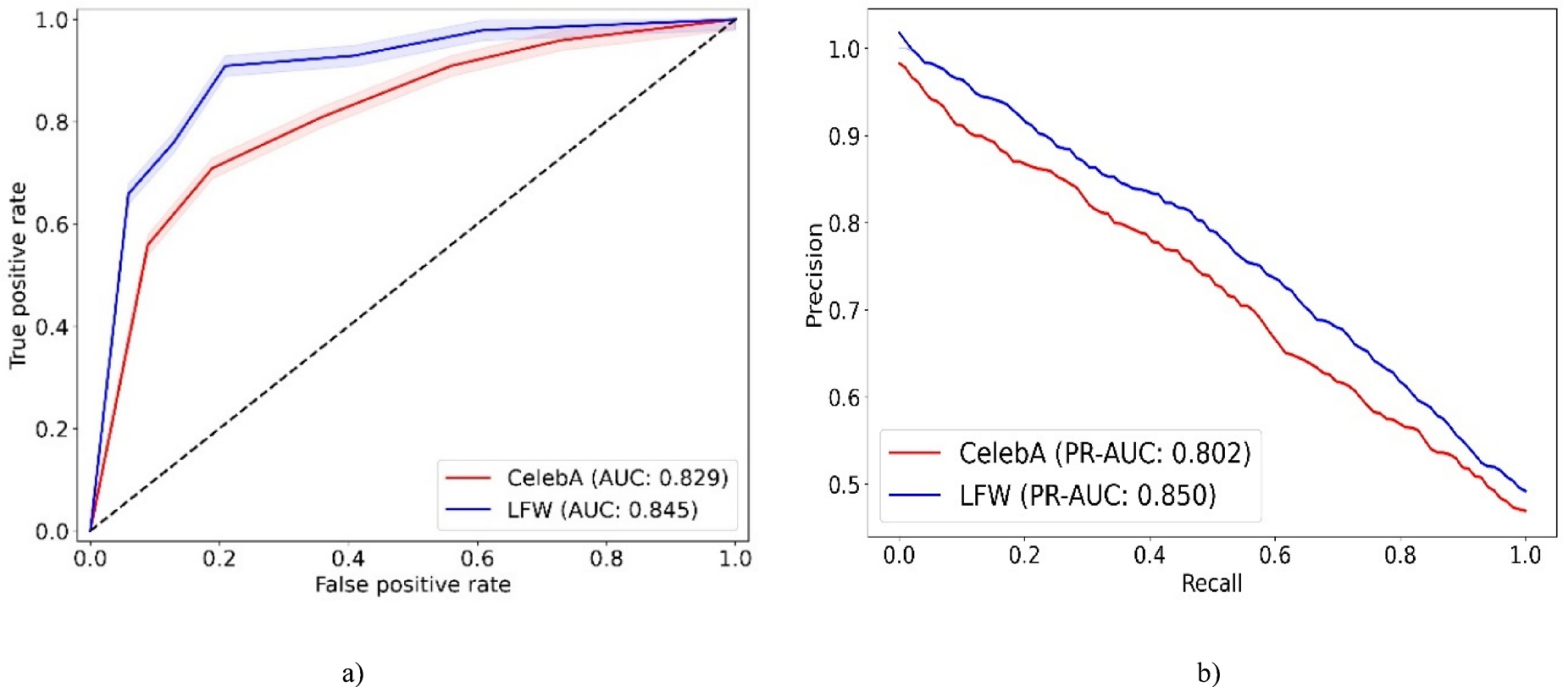
(**a**) ROC and (**b**) PR curves of the proposed model evaluated on the CelebA and LFW datasets.

Figure 11 presents the receiver operating characteristic (ROC) and precision-recall (PR) curves for the proposed model on the CelebA and LFW datasets. The ROC curves show that the model achieves AUC scores of 0.829 on CelebA and 0.845 on LFW. These values indicate that the model accurately detects positive cases. The PR curves reveal that the model performs well on imbalanced data, with PR-AUC scores of 0.802 for CelebA and 0.850 for LFW. Two main mechanisms contribute to this performance. First, Off-policy PPO dynamically selects features that are most useful for distinguishing between classes. Second, the reward function gives higher rewards to minority-class detections and stronger penalties to unnecessary majority-class selections. Together, these techniques maintain a favorable precision-recall balance, reduce the effects of class imbalance, and improve the effectiveness of the model in real-world criminal suspect identification.

**Fig. 12:**
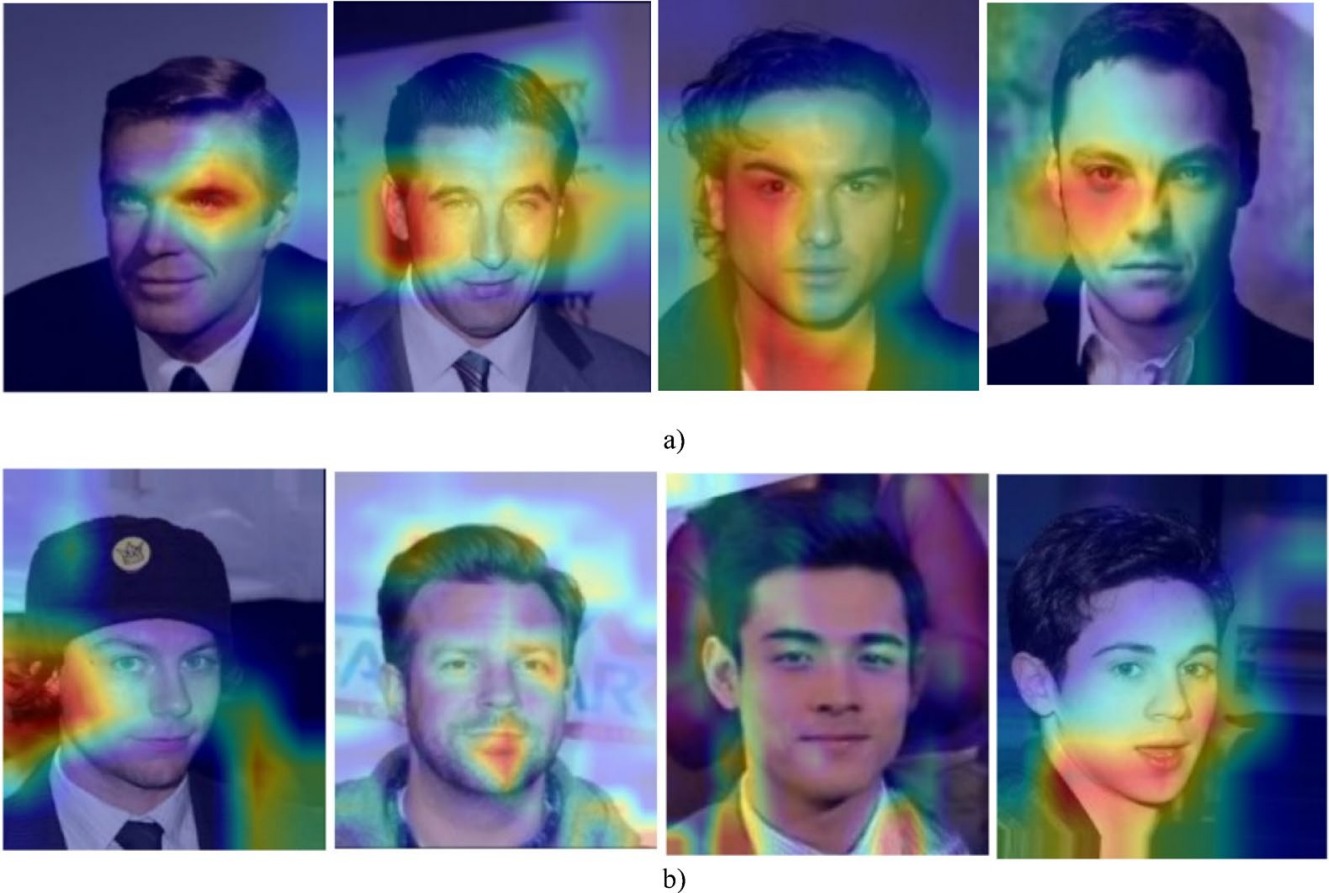
Visualization of feature extraction effectiveness using SHAP on key facial features for suspect identification across the (**a**) CelebA and (**b**) LFW datasets.

To improve interpretability and assess the model performance in feature extraction and selection, shapley additive explanations (SHAP) is used for visual analysis. Figure 12 shows the output results using the CelebA and LFW datasets. The model applies Off-policy PPO to dynamically choose the most informative features while handling class imbalance. Heatmaps generated by SHAP highlight the facial regions that have the most significant influence on the decisions of the model. Areas such as the eyes, nose bridge, lips, and upper cheeks consistently receive high importance scores. These regions are critical for suspect identification, as they tend to remain stable across different poses, lighting conditions, and facial expressions.

For CelebA, the heatmaps consistently highlight the periocular and mid-facial regions. This indicates that the model overlooks background elements, such as hair and clothing. Off-policy PPO is robust because it can focus on the most important features for classification. It does this even when there are variations within the same class, such as head tilts or different facial expressions. The LFW dataset includes real-world facial images with more background noise. On this dataset, the model focuses on the same facial regions as in CelebA. It also learns to use additional shape cues around the jawline and forehead. This flexibility demonstrates that the model can effectively manage challenging cases, including changes in lighting, occluded facial features, and cluttered scenes. These issues often happen in surveillance footage. Off-policy PPO uses dynamic FS and special rewards for minority classes. This helps the model avoid overfitting to the majority-class features. As a result, it pays balanced attention to important regions across all classes. The visual explanations demonstrate that the method aligns with human reasoning in forensic facial analysis. This enhances trust and makes the model more straightforward to interpret in real-world criminal identification settings.

#### Demo dataset

To demonstrate a simplified version of the proposed framework, we create a small dataset of 100 images from the CelebA dataset. This subset is used to visualize the pipeline of the method. It includes CNN-based feature extraction, PPO-driven FS, and final classification. Training a deep CNN from scratch on only 100 images is not practical. To address this, we use transfer learning. We pre-trained the CNN on the full CelebA dataset. Then, we fine-tune the learned weights on the smaller subset to preserve model reliability while showing the full process.

Table 15 shows step-by-step improvements in accuracy, F-measure, G-means, and AUC on these 100 samples. The first step, CNN feature extraction, acts as the baseline and uses standard classification. This setup achieves an accuracy of 84.26% and an AUC of 0.702. These results demonstrate that without FS or hyperparameter tuning, the model is unable to capture the fine variations in facial features that are useful for criminal identification.

**Table 15:** Stepwise performance improvements on the demo subset of the CelebA dataset.

Step	Accuracy	F-measure	G-means	AUC
Initial CNN	84.256	86.105	87.354	0.702
After FS (Off-policy PPO)	92.420	94.206	95.193	0.825
Final classification	96.102	98.635	99.565	0.919

After adding FS using PPO, the accuracy increases from 84.26% to 92.42%, representing a 9.16% improvement. AUC also rises from 0.702 to 0.825, about a 17.5% relative gain. This improvement results from dynamic FS using Off-policy PPO. It addresses class imbalance by rewarding detections of the minority class more and penalizing features that are irrelevant or redundant. As a result, the model creates a more useful feature space and reduces classification errors.

In the final stage, we include all three components: CNN feature extraction, PPO-based FS, and DE HO with cluster-guided mutation. Accuracy increases to 96.10% (a 4% gain over PPO alone), F-measure reaches 98.64%, and AUC improves to 0.919. This represents an 11.4% gain compared to the initial CNN step. These results demonstrate that DE optimization adjusts the model parameters to strike a balance between exploration and exploitation. This improves generalization and ensures stable training.

The progressive improvement across the three stages highlights the contribution of each component in the framework. CNN extracts the initial baseline features. Off-policy PPO improves the model by selecting more discriminative features and handling class imbalance. Finally, DE fine-tuning enables the model to reach an optimal configuration, thereby improving reliability in criminal suspect identification.

#### Analysis of the proposed DE

This section examines the effectiveness of the proposed DE algorithm in comparison to several widely used methods for hyperparameter tuning. The analysis is conducted with consistent model components across all evaluations. The study contrasts the proposed DE algorithm with four conventional search strategies, including random search, grid search, and Hyperband, Bayesian optimization (BO), and six established methods, including the salp swarm algorithm (SSA), HMS, cuckoo optimization algorithm (COA), firefly algorithm (FA), bat algorithm (BA), artificial bee colony (ABC), and original DE. Tables 16 and 17 present a comparative summary of HO results on the CelebA and LFW datasets.

**Table 16:** Comparative analysis of the proposed DE against other basic and metaheuristic optimization algorithms on the CelebA dataset.

Model	Accuracy	F-measure	G-means	AUC
Random search	67.511 ± 0.052	69.202 ± 0.046	70.929 ± 0.018	0.658 ± 0.066
Grid search	69.263 ± 0.095	71.753 ± 0.024	72.458 ± 0.026	0.662 ± 0.025
Hyperband	74.050 ± 0.037	76.855 ± 0.057	77.567 ± 0.036	0.672 ± 0.046
BO	80.869 ± 0.099	81.703 ± 0.086	82.412 ± 0.074	0.783 ± 0.023
SSA	72.314 ± 0.015	77.739 ± 0.079	78.428 ± 0.069	0.709 ± 0.059
HMS	73.476 ± 0.055	78.699 ± 0.085	79.397 ± 0.029	0.720 ± 0.029
COA	75.264 ± 0.042	80.000 ± 0.001	80.663 ± 0.099	0.736 ± 0.071
FA	76.976 ± 0.095	81.403 ± 0.043	82.091 ± 0.023	0.750 ± 0.075
BA	78.433 ± 0.024	82.610 ± 0.034	83.300 ± 0.075	0.756 ± 0.060
ABC	79.126 ± 0.049	83.604 ± 0.003	84.290 ± 0.011	0.771 ± 0.088
DE	80.147 ± 0.089	84.885 ± 0.053	85.585 ± 0.001	0.782 ± 0.023
Proposed DE	87.951 ± 0.096	89.409 ± 0.087	90.193 ± 0.030	0.829 ± 0.073

**Table 17:** Comparative analysis of the proposed DE against other basic and metaheuristic algorithms on the LFW dataset.

Model	Accuracy	F-measure	G-means	AUC
Random search	69.214 ± 0.049	71.389 ± 0.052	72.119 ± 0.081	0.665 ± 0.061
Grid search	70.767 ± 0.045	72.396 ± 0.066	73.158 ± 0.038	0.672 ± 0.001
Hyperband	76.507 ± 0.004	78.865 ± 0.009	79.618 ± 0.008	0.681 ± 0.084
BO	81.400 ± 0.010	83.935 ± 0.010	84.716 ± 0.013	0.792 ± 0.020
SSA	74.546 ± 0.091	80.403 ± 0.074	81.019 ± 0.038	0.722 ± 0.052
HMS	75.851 ± 0.003	81.628 ± 0.006	82.178 ± 0.095	0.731 ± 0.089
COA	77.299 ± 0.007	83.101 ± 0.074	83.628 ± 0.021	0.746 ± 0.075
FA	78.137 ± 0.095	84.180 ± 0.003	84.690 ± 0.096	0.763 ± 0.098
BA	79.575 ± 0.008	85.533 ± 0.026	86.056 ± 0.089	0.780 ± 0.041
ABC	81.243 ± 0.056	86.489 ± 0.090	87.046 ± 0.014	0.789 ± 0.002
DE	82.324 ± 0.083	88.023 ± 0.054	88.542 ± 0.080	0.800 ± 0.039
Proposed DE	89.141 ± 0.022	91.152 ± 0.042	92.718 ± 0.074	0.845 ± 0.045

Advanced metaheuristic algorithms produce better results by utilizing stochastic behavior and population-based search. ABC improves CelebA accuracy by 17.4%, and BA by 15.9%, compared to grid search. On the LFW dataset, ABC reaches 81.24%, which is 11.1% better than Hyperband. However, some methods, such as FA and COA, converge slowly and sometimes stagnate. The lack of targeted exploitation in high-quality regions of the hyperparameter space is the cause of these issues. The original DE also shows strong results, achieving 80.15% accuracy on CelebA and 82.32% on LFW. Still, it does not fully leverage interactions among parameters in specific clusters.

The proposed DE shows the highest performance in terms of both robustness and accuracy. On CelebA, it achieves an accuracy increases of 9.7% over the original DE, 7.8% over ABC, and 18.2% over Hyperband. It also improves G-means by 5.4% and AUC by 4.7%. On LFW, it achieves gains of 8.3% over DE and 6.6% over ABC, with improvements in G-means and AUC exceeding 5.3% and 4.5%, respectively. Its success comes from a cluster-based mutation strategy. This strategy uses k-means clustering to identify promising areas in the solution space, allowing both broad exploration and focused refinement. As a result, the proposed method avoids local minima and accelerates convergence, enabling robust hyperparameter tuning for improved criminal suspect identification.

The statistical significance of the proposed DE algorithm is evaluated using paired t-tests on the CelebA and LFW datasets. On the CelebA dataset, the proposed DE outperforms the strongest baseline, original DE, with statistically significant gains across all metrics. The p-values are 0.003 for accuracy, 0.004 for F-measure, 0.005 for G-means, and 0.002 for AUC. The associated 95% confidence intervals are [4.3%, 6.1%] for accuracy, [3.9%, 5.8%] for F-measure, [3.8%, 6.0%] for G-means, and [4.5%, 6.3%] for AUC. Similarly, on the LFW dataset, the proposed DE method significantly outperforms ABC, the strongest metaheuristic competitor. The p-values are 0.002 for accuracy, 0.003 for F-measure, 0.004 for G-means, and 0.003 for AUC. The corresponding 95% confidence intervals are [4.1%, 6.0%] for accuracy, [4.0%, 6.2%] for F-measure, [4.2%, 6.1%] for G-means, and [4.4%, 6.3%] for AUC. These low p-values and narrow confidence intervals indicate that the observed performance improvements are statistically significant and unlikely due to random variation. Furthermore, similar results with p-values under 0.01 and confidence intervals within the [4%, 6%] range are obtained when comparing the proposed DE with other basic and metaheuristic optimization methods. This demonstrates the consistent superiority of the proposed approach across both datasets.

**Fig. 13:**
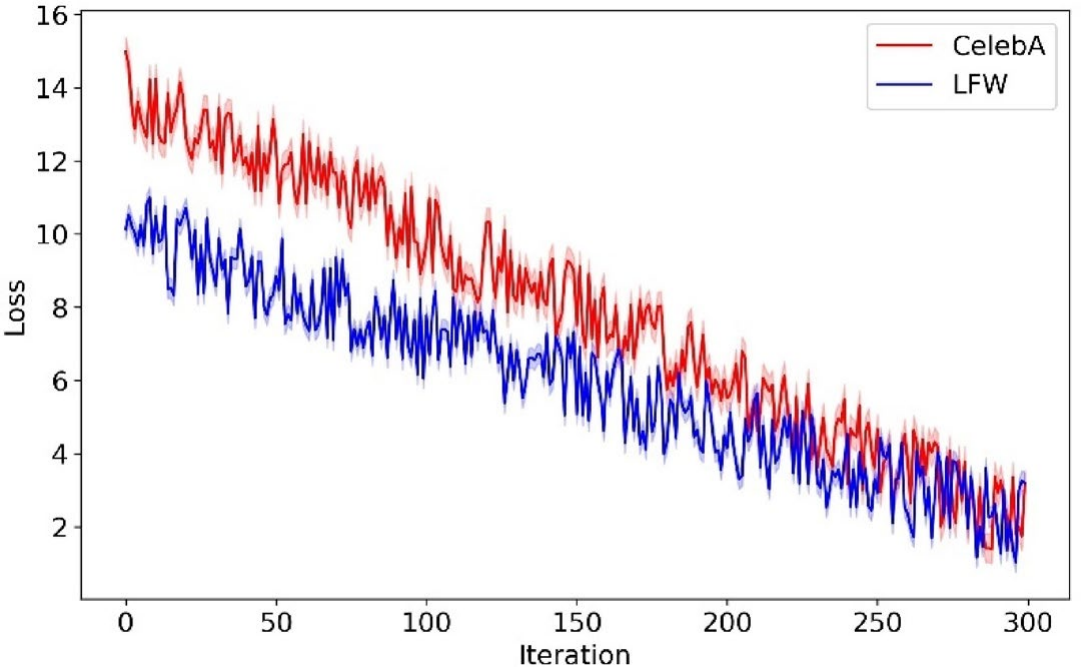
Loss reduction curves for the proposed DE algorithm over 300 iterations on the CelebA and LFW datasets.

Figure 13 illustrates the effectiveness of the proposed DE algorithm in optimizing hyperparameters across 300 iterations for both CelebA and LFW datasets. The graph shows a consistent decline in loss, confirming the capability of the algorithm to efficiently minimize errors and enhance model accuracy. Notably, the LFW dataset demonstrates a smoother and quicker reduction in loss compared to CelebA, suggesting the particular effectiveness of the algorithm in environments with varying data complexities. This shows that DE not only robustly handles a variety of datasets but also excels in adapting to different data characteristics, effectively avoiding overfitting. Such adaptability is crucial in real-world applications where datasets vary significantly, underscoring the potential of the proposed DE algorithm to enhance predictive performance and reliability in dynamic settings.

**Fig. 14:**
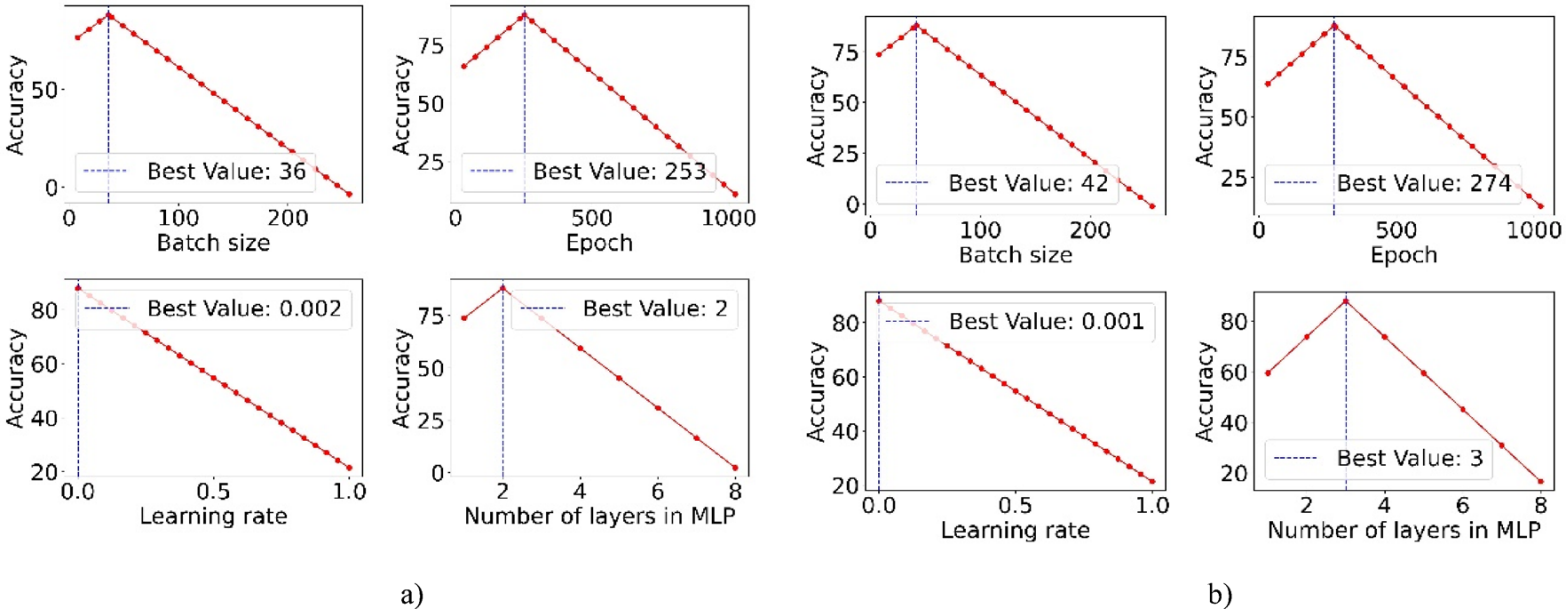
Effect of DE-based hyperparameter tuning on model accuracy across the (**a**) CelebA and (**b**) LFW datasets.

Figure 14 shows how the proposed DE method improves hyperparameter tuning for the model on the CelebA and LFW datasets. Each sub-figure illustrates how model accuracy varies with changes in batch size, epoch count, learning rate, and the number of layers in the MLP. On CelebA, DE identifies the optimal configuration with a batch size of 36, learning rate of 0.002, and 2 MLP layers, resulting in a peak accuracy improvement of approximately 12–15% over suboptimal settings. On LFW, the optimal hyperparameters include a batch size of 42, a learning rate of 0.001, and 3 MLP layers, which boosted performance by 10–14%. These results show that the proposed DE method avoids getting stuck in local optima. It also provides accurate hyperparameter tuning that improves both model accuracy and generalization.

#### Computational scalability and runtime analysis

**Fig. 15:**
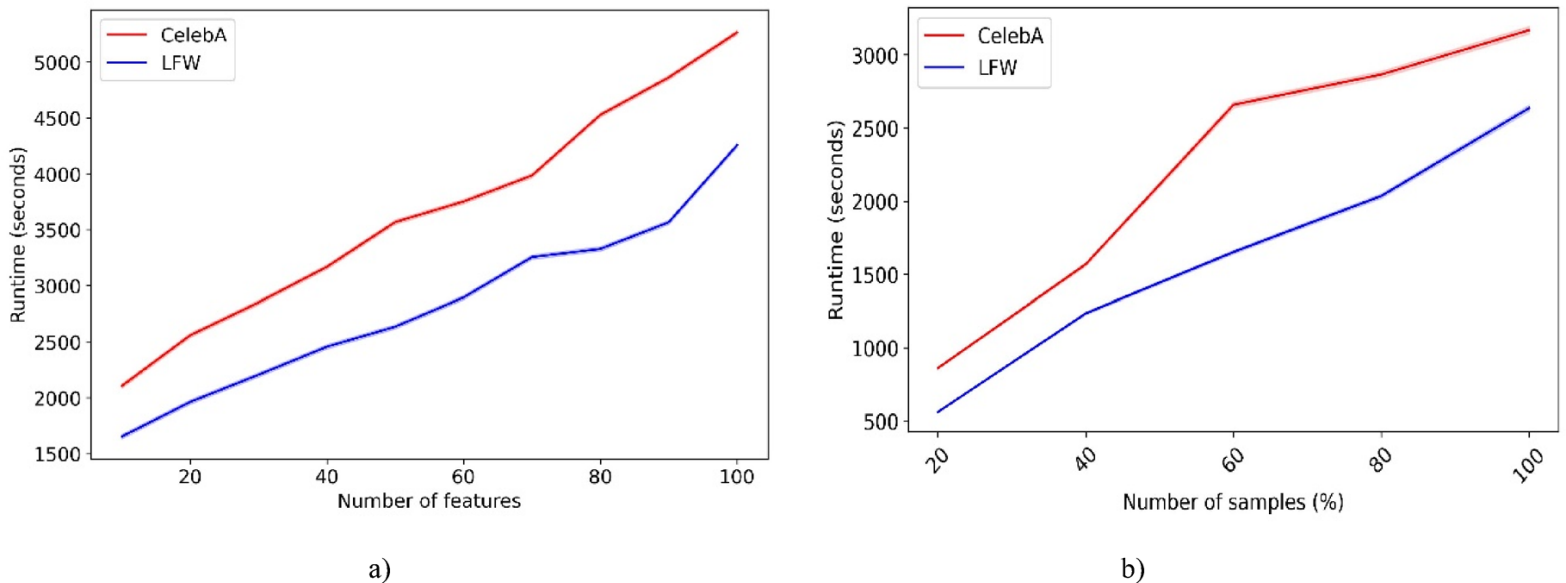
Runtime scalability analysis of (**a**) the number of selected features, and (**b**) the percentage of training samples, for the proposed framework using CelebA and LFW datasets.

Figure 15 illustrates the variation in the runtime of the proposed framework as the number of selected features and training samples changes, using the CelebA and LFW datasets. The runtime curve for FS shows that the runtime increases almost linearly as the number of selected features grows from 10 to 100. A larger feature set increases the search space for Off-policy PPO and DE, making FS and hyperparameter tuning more time-consuming. CelebA exhibits slightly higher runtimes than LFW because of its larger size and greater diversity of facial attributes. The runtime curve corresponding to the percentage of training samples shows how the runtime grows with the percentage of training samples. As the sample size increases from 20 to 100%, the runtime rises sharply, by about 220% on CelebA and 300% on LFW. This reflects the added computational burden from CNN feature extraction, PPO selection, and DE optimization. These results confirm that the framework is computationally scalable. However, the runtime increases in proportion to both the number of features and the size of the training data. This highlights the need for efficient optimization in large-scale scenarios.

Table 18 presents the runtime breakdown of the proposed framework across its three primary components: CNN feature extraction, Off‑policy PPO-based FS, and DE hyperparameter tuning on the CelebA and LFW datasets. CNN feature extraction takes the largest share of runtime, 62% for CelebA and 55% for LFW, because processing high-dimensional facial images and generating feature maps requires significant computational effort. The Off‑policy PPO FS accounts for 25% of the runtime on CelebA and 26% on LFW, due to the iterative process of evaluating feature subsets for both accuracy and class balance. DE hyperparameter tuning is the least time-consuming step, 13% for CelebA and 19% for LFW, but remains essential for achieving optimal model performance. This breakdown highlights that while CNN processing is the bottleneck, the integration of PPO and DE remains computationally efficient, supporting the scalability of the framework for larger datasets and real-world applications.

**Table 18:** Runtime distribution of the proposed framework key components on the CelebA and LFW datasets.

Step	Dataset			
	CelebA		LFW	
	Runtime (s)	% of Total	Runtime (s)	% of Total
CNN feature extraction	1964	62	1449	55
Off-policy PPO FS	792	25	685	26
DE hyperparameter tuning	412	13	501	19

#### Discussion

In this paper, a model is presented for the identification of criminal suspects. It offers two key advantages: (1) Off-policy PPO enables dynamic and effective FS while addressing class imbalance, and (2) the enhanced DE algorithm efficiently tunes hyperparameters. Together, these components improve both the accuracy and computational efficiency of suspect identification. The effectiveness of the model is validated on four publicly available datasets: CelebA, LFW, CASIA-WebFace, and VGGFace2. These datasets have been widely used for academic research in facial recognition and are distributed under licenses that permit non-commercial scientific use. CelebA and LFW contain images of public figures or individuals who have provided consent, and do not include personally identifiable information beyond facial images. CASIA-WebFace and VGGFace2 were collected explicitly for research purposes, and are publicly available for non-commercial academic work. Our use complies fully with the terms and ethical guidelines established by the dataset providers. Importantly, the study is conducted solely for academic purposes and does not involve real-world law enforcement, deployment, or decision-making. The model application in this context is entirely theoretical and exploratory in nature.

Integrating Off-policy PPO into the proposed model addresses two critical challenges: dynamic FS and class imbalance. Traditional methods often overlook complex, variable patterns essential for suspect identification and tend to favor majority classes. Off-policy PPO enables the model to repeatedly select the most useful features by utilizing long-term feedback from the environment. More importantly, it addresses class imbalance by assigning higher rewards or penalties to minority class samples, thereby encouraging the model to focus more on underrepresented cases. This strategy ensures that rare but important patterns are preserved and learned effectively. By reusing data from past interactions, the method enhances learning stability, reduces feature redundancy, and improves both accuracy and fairness in classification.

This study used an enhanced DE algorithm to optimize the hyperparameters of complex architectures such as Off-policy PPO. Unlike standard methods that often converge early or become stuck in local minima, DE employs a stochastic, population-based search strategy that thoroughly explores the parameter space. The process is further improved by a mutation strategy based on k-means clustering, which identifies parameter clusters that strongly impact performance. This targeted approach accelerates convergence and enhances the robustness of the model to data variation, thereby maintaining strong performance across diverse datasets. The study employs a single-objective fitness function based on classification accuracy, since accuracy is the main criterion for suspect identification. However, expanding this to a multi-objective framework that balances accuracy, computational efficiency, and model complexity could be a valuable direction for future work.

The proposed model combines theoretical innovation with real-world practicality. Theoretically, it integrates Off-policy PPO to address dynamic FS and class imbalance. In real scenarios, facial attributes are not static; lighting, pose, and environment constantly change. Off-policy PPO adaptively selects the most relevant features over time. It also gives stronger learning signals to minority classes, which traditional models often overlook. This ensures the model learns from rare but critical patterns. The enhanced DE algorithm automatically tunes hyperparameters, making the model easier to deploy in environments with limited computational resources. From a practical standpoint, the model is rigorously evaluated on established datasets, such as CelebA, LFW, CASIA-WebFace, and VGGFace2, consistently demonstrating improvements in accuracy, robustness, and computational efficiency compared to existing methods. This strong synergy between theoretical rigor and real-world validation confirms that the model is suitable for deployment in high-impact domains such as automated criminal suspect identification.

The proposed model demonstrates significant potential for real‑life applications, especially in enhancing public safety and supporting smaller industries or agencies with limited computational resources. The model combines Off‑policy PPO for dynamic FS and class imbalance handling with the enhanced DE algorithm for hyperparameter tuning. This integration achieves high accuracy and robustness while maintaining low computational demands. This efficiency is particularly beneficial for small law enforcement agencies, private security firms, and forensic laboratories that lack access to large GPU clusters or extensive technical expertise. The model can be integrated into lightweight surveillance systems to enable automated identification of potential suspects from security camera footage. This reduces the need for extensive manual review. Moreover, its adaptability allows deployment in scenarios where data collection is costly or limited, offering scalability to various operational environments. By providing a practical, accurate, and resource‑friendly solution, the model bridges the gap between advanced AI research and its tangible impact on real‑world security and investigative processes.

The methodology developed in this research has broad applicability beyond the identification of criminal suspects. For example, it can be effectively adapted for use in other areas where pattern recognition is critical, such as biometric authentication, surveillance, and social media analytics. The dynamic FS enabled by Off-policy PPO is particularly advantageous in environments where the input data is highly variable or rapidly evolving. Similarly, the ability of the enhanced DE algorithm to fine-tune hyperparameters makes the model adaptable to tasks requiring high precision, such as facial recognition in security systems or user identification in financial services. By implementing these methodologies, organizations can significantly improve the accuracy and efficiency of their pattern recognition systems, ensuring they remain effective even as new data challenges arise.

The limitations of the proposed model are as follows:

- *Data quality and error risk*: The effectiveness of the proposed model depends heavily on the quality and diversity of the input data. Noise, imbalance, or inherent bias in the dataset may affect both FS and classification. These issues increase the risk of misclassification. In criminal suspect identification, this may result in false positives, wrongly implicating innocent individuals, and false negatives, allowing actual suspects to go undetected. These errors carry serious social and legal consequences. These risks can be mitigated using several strategies, including data preprocessing, outlier removal, and bias detection methods such as fairness metrics and adversarial debiasing. Additionally, incorporating human-in-the-loop verification for low-confidence predictions and continuously updating datasets with diverse, real-world samples can significantly improve accuracy and fairness. Future work could explore integrating multimodal data, such as video sequences or contextual cues, to further reduce the risk of costly errors.
- *Model evolution and adaptability*: While Off-policy PPO enables dynamic FS within the current data distribution, it is not designed to accommodate gradual shifts in data over time. As a result, although the proposed framework demonstrates strong performance on the evaluated datasets, its long-term adaptability to evolving criminal behaviors and environmental variations remains a challenge. Over time, criminal strategies and facial characteristics may change. This leads to concept drift, where previously learned features no longer accurately predict outcomes. Without systematic updates, the accuracy of the model may degrade. Future implementations can improve robustness by using online or incremental learning. These techniques update model parameters with minimal retraining, helping the system adapt over time. Ensemble techniques can also enhance adaptability by maintaining multiple sub-models, each trained on a different data distribution. Proactive maintenance is essential. This includes regular retraining with new data, automated monitoring of accuracy, and scalable hyperparameter tuning. Real-time feedback from law enforcement agencies can guide updates and corrections. Additionally, community-driven datasets help the model remain relevant as new threats emerge. This continuous evolution ensures sustained operational performance in sensitive forensic and security applications.
- *Delayed adaptation in streaming environments*: Streaming environments involve continuous changes in incoming data. In such settings, the RL component (Off-policy PPO) may not adapt quickly to sudden changes in data distribution. This lag, especially during concept drift or abrupt shifts, can cause temporary drops in accuracy. Real-time systems require immediate responses. Any delay in adaptation can compromise performance in critical situations, such as live surveillance or the rapid identification of suspects. To address this, online learning modules can be added to the PPO framework. These modules support incremental updates without retraining the full model. Meta-learning strategies can also help by allowing the model to adjust quickly using only a few new examples. In addition, techniques such as experience replay buffers that prioritize recent data, adaptive learning rate schedules, and early drift detection can further improve response time. These enhancements support accurate and stable performance under dynamic conditions, enabling effective use in real-time forensic and security systems.
- *Limited generalizability to non-facial data*: The proposed model is evaluated using facial datasets, including CelebA, LFW, CASIA-WebFace, and VGGFace2. These datasets are clean, well-labeled, and collected in controlled environments. However, the model may not perform well in more chaotic and unstructured settings, such as low-quality CCTV footage, recordings from body-worn cameras, or multimodal forensic data. These real-world inputs often include occlusion, poor lighting, motion blur, and inconsistent resolution. As a result, the model can struggle when exposed to unfamiliar types of data. To improve generalizability, future research can explore domain adaptation and transfer learning. These techniques enable the model to utilize the knowledge it has gained from facial data and apply it to other data types. Adding data augmentation strategies that mimic real-world distortions and building training datasets with noise, occlusion, and varied environments can also improve robustness. Additionally, contrastive learning or self-supervised pretraining may help the model develop strong and transferable internal representations.

## Conclusion

This study successfully demonstrated the substantial benefits of integrating advanced RL techniques, specifically Off-policy PPO, with a refined DE algorithm to enhance the performance of CNNs in criminal suspect identification. By tackling critical issues such as FS, class imbalance, and HO, the proposed model significantly outperformed conventional methods, which frequently struggle with these challenges. Implementing Off-policy PPO allowed for dynamic adjustments in FS and class balancing, significantly diminishes the dependency of the model on large datasets, which are typically a constraint in real-world settings. Furthermore, the improved DE algorithm, which included a k-means clustering-based mutation strategy, effectively optimized the hyperparameters of the model. Empirical evaluations on the CelebA, LFW, CASIA-WebFace, and VGGFace2 datasets yielded remarkable results, with an F-measure above 89%. These results confirm the robustness and reliability of the model, demonstrating its exceptional ability to generalize across different datasets. These innovations highlight the potential of the model to transform suspect identification processes within legal frameworks, ensuring more precise and expedited justice. The outcomes of this research are poised to serve as a foundational element for future advancements for applying ML in criminal justice and other critical areas, fostering both technological progress and ethical accountability.

To support practical deployment, we acknowledge several challenges and outline directions for future developments. In future work, we plan to extend our model toward real-time data analysis and deployment in practical surveillance systems. One key direction involves integrating streaming data from live video feeds, enabling the system to deliver immediate suspect identification and predictive insights during ongoing investigations. To achieve this, several deployment challenges must be addressed. These include maintaining low-latency inference, adapting the model for low-power devices, such as mobile phones or edge systems, and ensuring stable performance under varying lighting conditions, motion blur, or occlusions in live video. We also plan to use multiple data types in the learning process, including witness descriptions, behavioral patterns, and audio input. This would allow a richer, more context-aware representation of suspect profiles. Leveraging advances in natural language processing and multimodal fusion could further boost identification accuracy in complex or ambiguous scenarios.

## Data Availability

The datasets are available from the authors upon reasonable request.
